# Cell envelope polysaccharide modifications alter the surface properties and interactions of *Mycobacterium abscessus* with innate immune cells in a morphotype-dependent manner

**DOI:** 10.1128/mbio.00322-25

**Published:** 2025-03-14

**Authors:** Elena Lian, Juan M. Belardinelli, Kavita De, Arun Prasad Pandurangan, Shiva K. Angala, Zuzana Palčeková, Anna E. Grzegorzewicz, Josephine M. Bryant, Tom L. Blundell, Julian Parkhill, R. Andres Floto, William H. Wheat, Mary Jackson

**Affiliations:** 1 Mycobacteria Research Laboratories, Department of Microbiology, Immunology and Pathology, Colorado State University, Fort Collins, Colorado, USA; 2 Victor Phillip Dahdaleh Heart and Lung Research Institute, Trumpington, Cambridge, UK; 3 Cambridge Centre for AI in Medicine, University of Cambridge, Cambridge, UK; 4 Parasites and Microbes Programme, Wellcome Sanger Institute, Hinxton, UK; 5 Department of Veterinary Medicine, University of Cambridge, Cambridge, UK; 6 Cambridge Centre for Lung Infection, Royal Papworth Hospital, Cambridge, UK; 7 Molecular Immunity Unit, Department of Medicine, University of Cambridge, MRC Laboratory of Molecular Biology, Trumpington, Cambridge, UK; Washington University in St. Louis, St. Louis, Missouri, USA

**Keywords:** *Mycobacterium abscessus*, non-tuberculous mycobacteria, lipoarabinomannan, arabinogalactan, biofilm, morphotype, virulence, immunomodulation

## Abstract

*Mycobacterium abscessus* is one of the leading causes of pulmonary infections caused by non-tuberculous mycobacteria. The ability of *M. abscessus* to establish a chronic infection in the lung relies on a series of adaptive mutations impacting, in part, global regulators and cell envelope biosynthetic enzymes. One of the genes under strong evolutionary pressure during host adaptation is *ubiA*, which participates in the elaboration of the arabinan domains of two major cell envelope polysaccharides: arabinogalactan (AG) and lipoarabinomannan (LAM). We here show that patient-derived UbiA mutations not only cause alterations in the AG, LAM, and mycolic acid contents of *M. abscessus* but also tend to render the bacterium more prone to forming biofilms while evading uptake by innate immune cells and enhancing their pro-inflammatory properties. The fact that the effects of UbiA mutations on the physiology and pathogenicity of *M. abscessus* were impacted by the rough or smooth morphotype of the strain suggests that the timing of their selection relative to morphotype switching may be key to their ability to promote chronic persistence in the host.

**IMPORTANCE:**

Multidrug-resistant pulmonary infections caused by *Mycobacterium abscessus* and subspecies are increasing in the U.S.A. and globally. Little is known of the mechanisms of pathogenicity of these microorganisms. We have identified single-nucleotide polymorphisms (SNPs) in a gene involved in the biosynthesis of two major cell envelope polysaccharides, arabinogalactan and lipoarabinomannan, in lung-adapted isolates from 13 patients. Introduction of these individual SNPs in a reference *M. abscessus* strain allowed us to study their impact on the physiology of the bacterium and its interactions with immune cells. The significance of our work is in identifying some of the mechanisms used by *M. abscessus* to colonize and persist in the human lung, which will facilitate the early detection of potentially more virulent clinical isolates and lead to new therapeutic strategies. Our findings may further have broader biomedical impacts, as the *ubiA* gene is conserved in other tuberculous and non-tuberculous mycobacterial pathogens.

## INTRODUCTION

Once considered an environmental saprophyte, *Mycobacterium abscessus* has steadily progressed over the last five decades to become one of the leading non-tuberculous mycobacteria causing chronic pulmonary infections around the world (1). Immunocompromised people and those with structural lung diseases such as chronic obstructive pulmonary disease, cystic fibrosis (CF), and non-CF bronchiectasis are particularly at risk (2–4). Unraveling how *M. abscessus* adapts to and persists in the human host is important to the development of optimized strategies to better control and treat these infections. One approach taken by our laboratories and others to gain insight into the pathoadaptation of *M. abscessus* has consisted of tracking adaptive mutations in serially isolated *M. abscessus* clinical isolates from patients with pulmonary disease using partial or whole-genome sequencing (5–8). Among the loci found repeatedly mutated in multiple patients at a higher rate than would be expected by chance were genes involved in glycopeptidolipid (GPL) biosynthesis, lipoglycan biosynthesis (*embC*), response to iron starvation and redox stress (*ideR* and *whiB1*), and global regulation (*phoR* and *crp*) (5–7). Loss-of-function mutations in GPL biosynthetic genes result in a smooth-to-rough morphotype transition that accompanies changes in the cording properties and biofilm-forming capacity of the bacterium. Rough (R) (GPL-deficient) variants are associated with disease progression and clinical decline and are characterized by the ability to withstand phagocytosis and increase macrophage apoptosis and a “hyper-pro-inflammatory” TLR-2-dependent response (9–16). Recent studies from our laboratory indicated that patient-derived mutations in *phoR* and *embC* similarly have important impacts on the physiology of the bacterium and its interaction with the host. In the case of the sensor kinase gene *phoR*, the mutations manifest by an exacerbation of the response of *M. abscessus* to acidic pH, leading to the upregulation of genes involved in host adaptation (17). In the case of *embC*, mutations alter the structure of a major cell envelope lipoglycan known as lipoarabinomannan (LAM) and further impact the cording, biofilm formation, sliding motility, and immunomodulatory properties of the bacterium (18).

**Fig 1:**
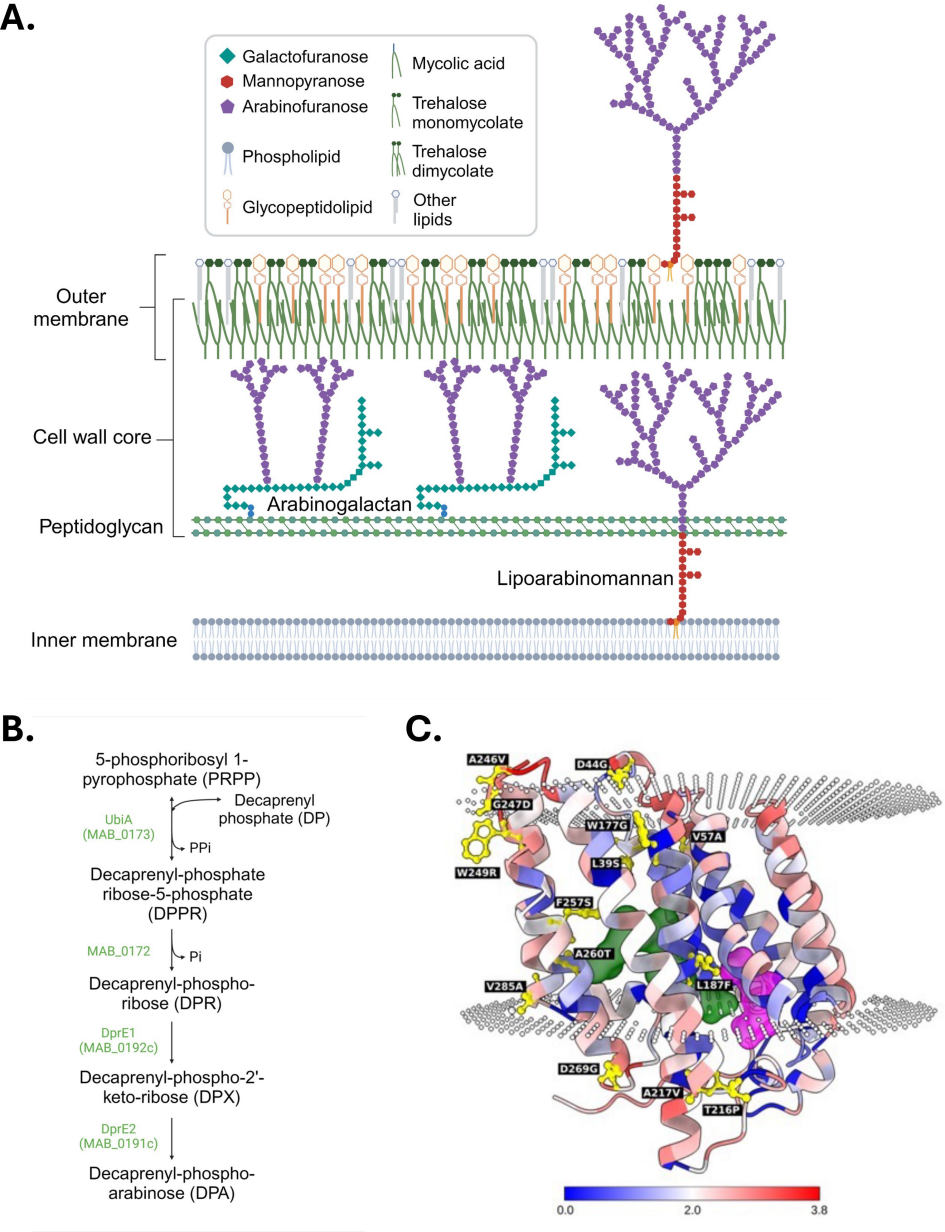
Schematic representations of the *M. abscessus* cell envelope and decaprenyl-phosphate-arabinose (DPA) biosynthetic pathway and analysis of the patient-derived UbiA mutations. (**A**) Schematic representation of the cell envelope of *M. abscessus*. The lipids and glycolipids mentioned within this paper are shown in the schematic. Proteins are not shown for clarity. The depicted structures are not drawn to scale. Created in BioRender. (**B**) DPA biosynthesis. DPA is the only known arabinose donor used by mycobacterial arabinosyltransferases to elongate the arabinan domains of AG and LAM. It is synthesized from 5-phosphoribosyl-1-pyrophosphate (PRPP) through a four-step reaction involving the decaprenyl-phosphate 5-phosphoribosyltransferase UbiA, a phosphoribosyl monophosphodecaprenol phosphatase, and the heteromeric DprE1/DprE2 responsible for the epimerization of decaprenyl-phosphate-ribose to DPA (29). Created in BioRender. (**C**) AlphaFold model of UbiA. The 14 patient-derived mutations are labeled and shown as a ball-and-stick model in yellow. The ligands, DP and PRPP, are shown as surfaces in green and magenta, respectively. The residue conservation, based on the UbiA family alignment extracted from the CDD database, is color-coded on the structure from blue (high conservation) to red (low conservation), and the corresponding color bar is shown at the bottom. This analysis shows that the core residues of the transmembrane helices are more conserved compared to the cytoplasmic and periplasmic residues. The orientation of UbiA within the membrane bilayer was predicted using the OPM program. The regions above the upper dotted disk and below the lower dotted disk represent the periplasmic and cytoplasmic regions, respectively.

*ubiA*, a cell envelope biosynthetic gene, is another gene that was found to be mutated at a rate higher than expected by chance in *M. abscessus* isolates from multiple infected CF patients (5). The fact that *ubiA*, along with GPL biosynthetic genes and global regulators (*phoR*, *engA*, and *crp*), was identified as one of the top four loci accumulating non-synonymous single-nucleotide polymorphisms (NS SNPs) in the course of infection points to *ubiA* mutations as one of the preferred mechanisms of adaptation of *M. abscessus* to infection (5). Furthermore, the fact that NS mutations in *ubiA* occurred at a significantly lower rate in laparoscopy-associated *M. abscessus* wound infections than observed during pulmonary disease suggests that *ubiA* mutations may confer a particular advantage to the bacterium during lung infection (5). The UbiA enzyme initiates the biosynthetic pathway for decaprenyl-phosphate-arabinose (DPA), which is the only known arabinose donor used by mycobacteria to synthesize arabinogalactan (AG) and LAM, two major cell envelope glycans (19–21). AG is essential to the integrity of the bacterium. Together with peptidoglycan, to which it is covalently attached, AG makes up the bulk of the cell wall core and provides a rigid structure outside the plasma membrane that serves as a scaffold for the rest of the cell envelope (22) (Fig. 1A). LAM is anchored through its phosphatidyl-*myo*-inositol lipid tail in both the inner and outer membranes, where it contributes to the structural integrity of the cell envelope and the modulation of host immune responses to infection (23–28) (Fig. 1A). To determine whether mutating *ubiA* may confer upon *M. abscessus* an adaptive advantage for survival in the host, we characterized here a subset of clinically relevant *ubiA* mutations in terms of their functional impact on the bacterium and their interaction with human innate immune cells. Because *ubiA* mutations have been reported to occur in both smooth (S) (GPL-proficient) and rough (GPL-deficient) isolates (5, 7), these analyses were conducted in both morphotypes.

## RESULTS

### Mutational variations in the *ubiA* gene of *M. abscessus* isolates recovered from the lung of long-term infected patients

Whole-genome sequencing of serial isolates from 201 CF patients (30) identified *ubiA* (*MAB_0173*) as one of the genes accumulating more NS SNPs than would be expected by chance in as many as 13 patients (5). *ubiA* encodes a decaprenyl-phosphate 5-phosphoribosyl transferase and is the first committed gene in the biosynthesis of DPA, the arabinose donor used in the synthesis of AG and LAM (Fig. 1B). *ubiA* mutations were present in all three *M. abscessus* subsp. (*M. abscessus* subsp. *abscessus* [*n* = 4], *M. abscessus* subsp. *massiliense* [*n* = 7], and *M. abscessus* subsp. *bolletii* [*n* = 2]). Of note, one codon (at position 260) was mutated in two independent patients, and two of the mutations were found in two hypermutating strains.

Mapping of all 14 different NS SNPs found in UbiA from *M. abscessus* (L39S, D44G, V57A, L187F, T216P, W177G, A217V, A246V, G247D, W249R, A260T, F257S, D269G, and V285A) on the AlphaFold model of this protein indicated that the numbers of mutations predicted to be positioned in the membrane bilayer, cytoplasm, periplasm, and interface are 7, 3, 2, and 2, respectively (Fig. 1C). Eleven out of 14 mutations, including the four mutations studied experimentally herein (V57A, L39S, A260T, and T216P), were predicted to destabilize the protein by both SDM and FoldX (Table S1). The three mutations where the two programs disagreed (A217V, V285A, and W249R) were found to marginally stabilize or destabilize the protein by only ±1 kcal/mol.

Mutations V57A, L39S, and A260T are found within the bilayer in a tightly packed hydrophobic environment with occluded surface packing (OSP) density values of 0.60, 0.30, and 0.50, respectively (Fig. 1C, Table S1). Mutation T216P, in contrast, is located on the cytoplasmic face of the membrane and directly affects the N cap residue of the seventh α-helix (Fig. 1C). The N cap residue is the N-terminal residue of an α-helix that stabilizes it by forming a side-chain hydrogen bond with the main-chain carbonyl atom of the i + 3 residue from the same helix. A threonine residue is predominantly found at this position (31). The threonine to proline substitution abolishes the hydrogen bond and is likely to destabilize the helix.

All 14 mutations were otherwise predicted to be allosterically coupled to the decaprenyl phosphate (DP) and 5-phosphoribosyl-1-pyrophosphate (PRPP) binding sites (median allosteric coupling intensity [ACI] values of 0.50 and 0.45, respectively) and to reduce the binding affinity of DP and/or PRPP to UbiA. Only three mutations were predicted to reduce binding to DP more than to PRPP (D44G and D269G) or vice versa (L187F). All other mutations were predicted to similarly affect the binding of both substrates (Fig. S1).

### Generating isogenic mutants of *ubiA* in smooth and rough variants of *M. abscessus*

Of the 14 identified NS SNPs in the *ubiA* gene, four were selected for further study: L39S, V57A, T216P, and A260T. These single-nucleotide polymorphisms (SNPs) are located within the transmembrane (L39S, V57A, and A260T) or cytoplasmic (T216P) regions of the enzyme and predicted to affect to various degrees its stability and substrate-binding affinity (Fig. 1C; Table S1; Fig. S1). Of these mutations, codon 260 was found to be mutated in two independent patients. The functionality of the mutated UbiA variants (thereafter referred to as UbiA^L39S^, UbiA^V57A^, UbiA^T216P^, and UbiA^A260T^) was further assessed in both the R and S morphotypes to enrich our understanding of how the presence or absence of GPLs in the outer membrane may affect consequent phenotypes. Indeed, GPLs have been shown to mask underlying immunogenic components of the cell envelope from the immune system, resulting in a dampened inflammatory response (13, 14). Determining whether GPL impacted the presentation to innate immune cells of LAM and other cell envelope constituents directly or indirectly impacted by the UbiA mutations was thus of interest in the context of this study. Moreover, of the few clinical isolates for which morphotype information was available in the Bryant et al. study (5), the A260T mutation occurred in an R isolate, whereas an unspecified *ubiA* NS SNP was reported to have occurred in an S isolate in an independent study (7).

Isogenic mutant strains were constructed in the R and S variants of *M. abscessus* subsp. *abscessus* American Type Culture Collection (ATCC) 19977. Because *ubiA* is an essential gene in *M. abscessus*, the native *ubiA* gene was deleted by allelic replacement in merodiploid *M. abscessus* (R and S) strains expressing a wild-type (WT) copy of the *ubiA* gene (UbiA^WT^) from an integrative plasmid, as described under Materials and Methods. Next, integrative plasmids expressing the mutated variants of *ubiA* were transformed into the merodiploid strain, leading to the replacement of the *ubiA^WT^* gene by the mutated variants (Fig. S2).

### Mutations in *ubiA* do not enhance antibiotic resistance

The *ubiA* SNPs were originally identified in *M. abscessus* isolates from infected patients who were on long-term antibiotic treatment. To determine whether the mutations were associated with antibiotic resistance, we performed a susceptibility test against a variety of antibiotics, some of which are commonly used for treating *M. abscessus* infection (32). In 7H9-albumin-dextrose-catalase (ADC)-Tween 80 medium, any marked differences in minimum inhibitory concentrations (MICs) (≥4-fold) were associated with increased antibiotic susceptibility of the UbiA^T216P^ mutant generated in the S morphotype. UbiA^T216P^ (S) was eight-times more susceptible to rifampin and four-times more susceptible to rifabutin, linezolid, and vancomycin (Table 1; Table S2). We conclude from these results that the *ubiA* mutations were not selected in patients because of their drug-resistant phenotype.

**TABLE 1:** Susceptibility to antibiotics of smooth and rough *ubiA* mutant strains in 7H9-ADC-Tween 80^*a*^

Antibiotic	Smooth					Rough				
	WT (S)	L39S (S)	V57A (S)	T216P (S)	A260T (S)	WT (R)	L39S (R)	V57A (R)	T216P (R)	A260T (R)
Rifampin	4	2	2	0.5	4	2	1	1	1–2	1–2
Rifabutin	0.5–1.0	0.5	0.5	0.125	0.5	1	0.5	0.5	1	1
Vancomycin	4	2	2–4	1	2	1	0.5	0.5	1	0.5
Linezolid	4	2	2	1	2	2	1	2	1–2	1–2
Cefoxitin	4	4	4	4	4–8	4	2	4	8	2–4
Amikacin	16	16	16–32	16	16	8	4	4	8	4
Levofloxacin	2	1–2	1–2	2	2	2	1	1	2	1
Tigecycline	2	2	2	2	2	2	2	2	2–4	2
Ethambutol	32	32	32	16	16–32	32	16	32	32	16
Azithromycin	256	512	512	256	512	256	256	128	256	256

^*a*^ MIC values (μg/mL) were recorded at 5 days post-treatment for all compounds except for azithromycin. Results for the latter compound were recorded at 14 days post-treatment. The values reported are representative of two independent experiments.

### Impact of *ubiA* mutations on LAM

**Fig 2:**
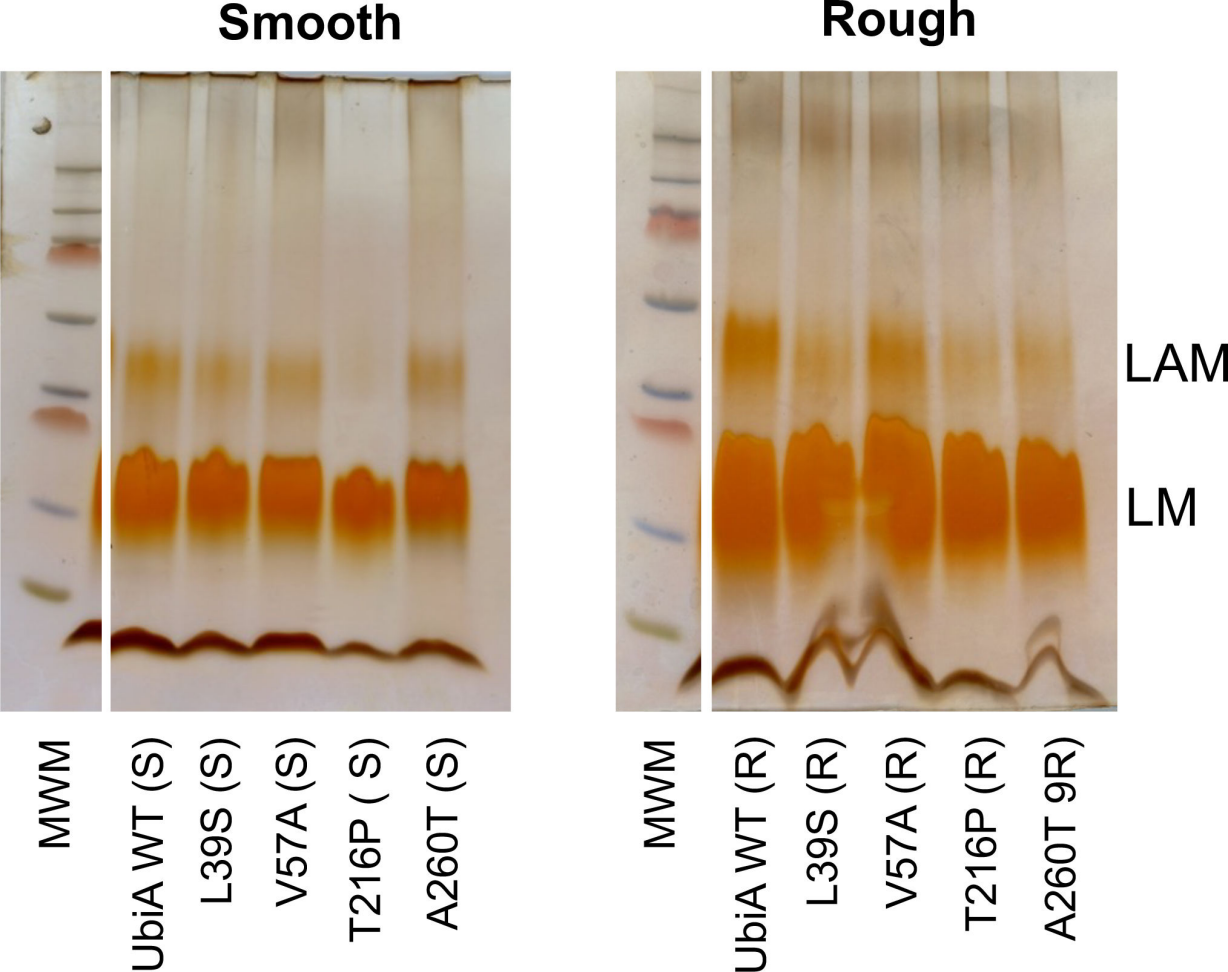
SDS-PAGE analysis of LAM from *M. abscessus* rough and smooth strains expressing different mutated variants of *ubiA*. Purified LAMs from the different strains were run on a 10%–20% tricine gel followed by periodic acid–silver staining. Equal volumes of lipoglycan preparations for each strain (standardized to the wet weight of bacterial cells) were loaded per lane. MWM, molecular weight marker.

To determine whether the patient-derived NS SNPs had any functional impact on the activity of UbiA, we first compared the LAM purified from the control R and S strains (UbiA^WT^ [R] and UbiA^WT^ [S]) to that purified from the corresponding isogenic mutants. Analysis of extracted lipoglycans by SDS-PAGE revealed very little LAM present for UbiA^T216P^ (S) and a notably decreased abundance in the UbiA^L39S^ (R), UbiA^T216P^ (R), and UbiA^A260T^ (R) mutants (Fig. 2). Analyses of per-*O*-methylated alditol acetate derivatives of the control and mutant LAMs were next undertaken to gain insight into the structures of their mannan and arabinan domains. While the Ara*f*-to-Man*p* ratios of LAM from mutants UbiA^L39S^, UbiA^T216P^, and UbiA^A260T^ were not significantly different from those of the controls in both morphotypes, UbiA^V57A^ (R) displayed a slightly reduced Ara*f*-to-Man*p* ratio (Table 2). Each mutation otherwise induced its own set of changes in LAM, which, interestingly, differed in the R and S backgrounds. This is particularly evident for the L39S mutation, which led to relatively minor changes in the structure of LAM in the R morphotype but to much more significant ones in the S morphotype (Table 2). Overall, the arabinan domains in the S morphotype LAMs were more severely altered than those in the R morphotype. This was characterized by a significant increase in the relative percentage of *t*-Ara*f* that accompanied a decrease in 3,5-linked Ara*f* (exception made of UbiA^V57A^ [S]) and an increase (in UbiA^V57A^ [S] and UbiA^A260T^ [S]) or a decrease (in UbiA^T216P^ [S]) in 5-linked Ara*f* (Table 2). Comparatively, the arabinan domain of the UbiA^V57A^ (R), UbiA^T216P^ (R), and UbiA^A260T^ (R) mutants only showed a significant decrease in 5-linked Ara*f*. In fact, structural changes in the LAM of R mutants mostly affected the mannan domain, which was significantly more branched in UbiA^V57A^ (R), UbiA^T216P^ (R), and UbiA^A260T^ (R) based on relative percentages of 3,6-linked Man*p* residues (Table 2). This phenotype is opposite to the mannan domains of all S mutants, which were, on the contrary, significantly less branched than the control WT LAM (Table 2). As observed previously in other *M. abscessus* mutants with altered arabinan domains (18), the presence of 3-linked oligomannosides significantly increased in two S mutants (UbiA^L39S^ [S)] and UbiA^T216P^ [S)] as well as in UbiA^A260T^ (R) but decreased in UbiA^L39S^ (R) (Table 2). Finally, analysis of the non-reducing arabinan termini of LAM upon *Cellulomonas gelida* endoarabinanase digestion also revealed morphotype-dependent changes caused by each mutation (Table S3). Overall, changes in the S mutants essentially manifested in the reduced acetylation of Ara_4_ and/or Ara_5_ termini, with the exception of UbiA^A260T^ (S), which showed more acetylated Ara_4_ and Ara_6_. Changes in the R mutants were more variable and mutation dependent. In both morphotypes, the UbiA^T216P^ and UbiA^A260T^ mutants were the ones whose LAM produced the smallest relative percentage of Ara_4_ termini relative to Ara_5_ and Ara_6_ (Table S3). Ultimately, each *ubiA* mutation resulted in its own specific alterations in the structure and abundance of LAM, which were influenced by the morphotype in which these mutations were expressed. While some consistency in the type of glycosyl linkages affected by multiple mutations in the R and S strains (e.g., 3-Man*p*, 3,6-Man*p*, *t*-Man*p*, and 5-Ara*f*) provides confidence in their relationship to changes in the UbiA protein, other less conserved and more discrete changes may be within the range of batch-to-batch variability one would expect in the structure of LAM and may not be biologically significant.

**TABLE 2:** Ara*f*/Man*p* ratio and glycosyl linkage analysis for per-*O*-methylated LAM from smooth and rough *ubiA* mutant strains^*a*^

Strain	*t*-Ara*f*	2-Ara*f*	5-Ara*f*	3,5-Ara*f*	*t*-Man*p*	3-Man*p*	6-Man*p*	3,6-Man*p*	Ara*f*/ Man*p*
Smooth									
WT (S)	1.4 ± 0.3	0.8 ± 0.3	35.5 ± 2.3	15.3 ± 1.1	9.1 ± 0.8	11.6 ± 1.2	6.6 ± 1.5	17.4 ± 5.7	2.0 ± 0.01
L39S (S)	8.3 ± 0.8****	1.0 ± 0.02	32.0 ± 1.4	10.9 ± 0.2****	11.4 ± 0.5**	26.6 ± 1.3****	5.3 ± 0.4	4.6 ± 0.8***	1.6 ± 0.3
V57A (S)	10.1 ± 0.4****	1.3 ± 0.1*	42.2 ± 1.4**	14.8 ± 0.01	11.0 ± 0.3**	9.1 ± 0.9*	5.7 ± 3.6	6.0 ± 1.4**	1.7 ± 0.1
T216P (S)	8.3 ± 1.0****	0.9 ± 0.1	28.6 ± 0.3***	8.8 ± 0.3****	11.4 ± 0.7**	33.7 ± 0.5****	4.4 ± 0.2	3.9 ± 0.8***	1.7 ± 0.3
A260T (S)	11.1 ± 1.3****	3.9 ± 0.3****	39.7 ± 1.6*	13.2 ± 0.3**	9.8 ± 0.1	9.6 ± 0.7	7.2 ± 0.3	5.6 ± 0.9**	2.0 ± 0.1
Rough									
WT (R)	11.5 ± 1.2	1.8 ± 0.3	41.8 ± 1.7	15.0 ± 0.6	8.3 ± 0.3	12.6 ± 0.9	4.8 ± 0.2	4.2 ± 0.5	2.3 ± 0.2
L39S (R)	12.9 ± 2.9	1.3 ± 0.4	36.3 ± 5.5	11.9 ± 2.0*	8.0 ± 0.6	6.7 ± 1.1***	5.6 ± 0.5	6.2 ± 0.8	2.1 ± 0.1
V57A (R)	13.2 ± 0.1	1.5 ± 0.3	28.6 ± 0.7**	14.5 ± 0.5	11.4 ± 0.8***	12.2 ± 0.9	9.7 ± 0.4***	9.2 ± 0.1***	1.8 ± 0.2*
T216P (R)	13.3 ± 0.2	3.1 ± 0.2**	28.5 ± 1.0**	14.9 ± 0.1	9.7 ± 0.1	12.8 ± 1.3	9.5 ± 0.8***	7.7 ± 0.2**	2.2 ± 0.2
A260T (R)	10.3 ± 0.7	2.1 ± 0.5	28.9 ± 4.5**	12.6 ± 0.7*	10.8 ± 1.0**	16.5 ± 0.9**	10.6 ± 1.7****	8.0 ± 2.5**	1.9 ± 0.2

^*a*^ Values are reported as the average ± SD of two to three technical replicates from one experiment and represent the relative distribution in percent. A one-way analysis of variance with Dunnett’s multiple comparison test was performed against UbiA WT (S or R) for each type of linkage and for the Araf/Manp ratio. *P < 0.05, **P < 0.01, ***P < 0.001, ****P < 0.0001.

### Impact of *ubiA* mutations on AG structure and mycolic acid distribution in the cell envelope

Analyses of per-*O*-methylated alditol acetate derivatives of the control and mutant mycolyl-AG-peptidoglycan (mAGPs) revealed fewer and quantitatively more minor changes in the structure of AG compared to LAM (Table 3). Similar to the situation with LAM, the mutations had a more profound effect on the arabinan domain of AG in the S strains than in the R strains. These changes were characterized by a decrease in the relative percentage of *t*-Ara*f* in all four S mutants that accompanied a mild increase in 3,5-linked Ara*f* (exception made of UbiA^A260T^ [S]). All four S mutants also presented a decrease in the relative percentage of 5,6-linked Gal*f* indicative of structural changes in the galactan domain as well (Table 3). These changes were not observed in the R mutants, which showed, on the contrary, an increase in the relative percentage of *t*-Ara*f* (UbiA^V57A^ [R] and UbiA^T216P^ [R]), a mild decrease in 3,5-linked Ara*f* (all four R mutants), and a slight increase in 6-linked Gal*f* (all four mutants) (Table 3). Despite these changes, the Ara*f*/Gal*f* ratio of all R and S mutant AGs remained largely unchanged, with the exception of UbiA^A260T^ (R), which displayed a significantly smaller ratio (Table 3). As in the case of LAM, some of the minor changes in the structure of AG outlined above may be within the range of batch-to-batch variability and may not be biologically significant.

**TABLE 3:** Ara*f*/Gal*f* ratio and glycosyl linkage analysis for per-*O*-methylated AG from smooth and rough *ubiA* mutant strains^*a*^

Strain	*t*-Ara*f*	2-Ara*f*	5-Ara*f*	3,5-Ara*f*	*t*-Gal*f*	5-Gal*f*	6-Gal*f*	5,6-Gal*f*	Ara*f*/Gal*f*
Smooth									
WT (S)	11.8 ± 0.1	4.4 ± 0.9	43.0 ± 1.9	10.1 ± 0.7	4.4 ± 0.9	14.4 ± 1.4	9.2 ± 0.5	2.6 ± 0.4	2.3 ± 0.3
L39S (S)	6.3 ± 0.9****	4.8 ± 0.3	44.2 ± 1.6	11.7 ± 0.3*	4.7 ± 1.1	16.9 ± 0.7	9.6 ± 0.3	1.8 ± 0.1*	2.0 ± 0.2
V57A (S)	7.5 ± 1.0***	4.8 ± 0.1	41.7 ± 1.4	12.0 ± 0.3**	5.6 ± 0.7	16.9 ± 0.5	9.7 ± 0.2	1.8 ± 0.1**	1.9 ± 0.03
T216P (S)	9.0 ± 0.5**	3.6 ± 0.5	44.6 ± 1.6	11.7 ± 0.5*	5.1 ± 0.4	15.1 ± 1.7	9.2 ± 0.8	1.8 ± 0.1*	2.2 ± 0.3
A260T (S)	9.2 ± 0.3*	6.4 ± 0.1**	44.9 ± 1.3	9.9 ± 0.4	4.9 ± 1.1	14.3 ± 0.5	8.5 ± 0.3	1.8 ± 0.1*	2.4 ± 0.2
Rough									
WT (R)	6.6 ± 1.2	5.3 ± 0.4	42.1 ± 0.5	12.8 ± 0.5	4.4 ± 0.4	17.5 ± 0.1	9.0 ± 0.4	2.3 ± 0.2	2.0 ± 0.1
L39S (R)	7.4 ± 1.2	3.1 ± 0.4***	42.2 ± 1.2	10.6 ± 0.1***	3.8 ± 0.6	19.3 ± 0.1	11.4 ± 0.5**	2.2 ± 0.1	1.7 ± 0.01
V57A (R)	10.1 ± 0.1*	3.8 ± 0.2**	41.8 ± 1.8	11.3 ± 0.2**	3.8 ± 0.7	16.5 ± 0.8	10.5 ± 0.7*	2.2 ± 0.1	2.0 ± 0.2
T216P (R)	10.1 ± 0.9*	5.0 ± 0.2	40.6 ± 0.7	10.5 ± 0.3***	4.1 ± 0.3	16.8 ± 0.9	10.5 ± 0.5*	2.5 ± 0.3	2.0 ± 0.1
A260T (R)	6.5 ± 1.0	4.9 ± 0.2	38.7 ± 0.8*	10.6 ± 0.2***	5.6 ± 0.4	20.4 ± 0.6**	11.1 ± 0.3**	2.2 ± 0.1	1.5 ± 0.1**

^*a*^ Values are reported as the averages ± SDs of two to three technical replicates from one experiment and represent the relative distribution in percent. A one-way analysis of variance with Dunnett’s multiple comparison test was performed against UbiA WT (R/S) for each type of linkage and for the Ara*f*/Gal*f* ratio: *P < 0.05, **P < 0.01, ***P <0.001, ****P < 0.0001.

**Fig 3:**
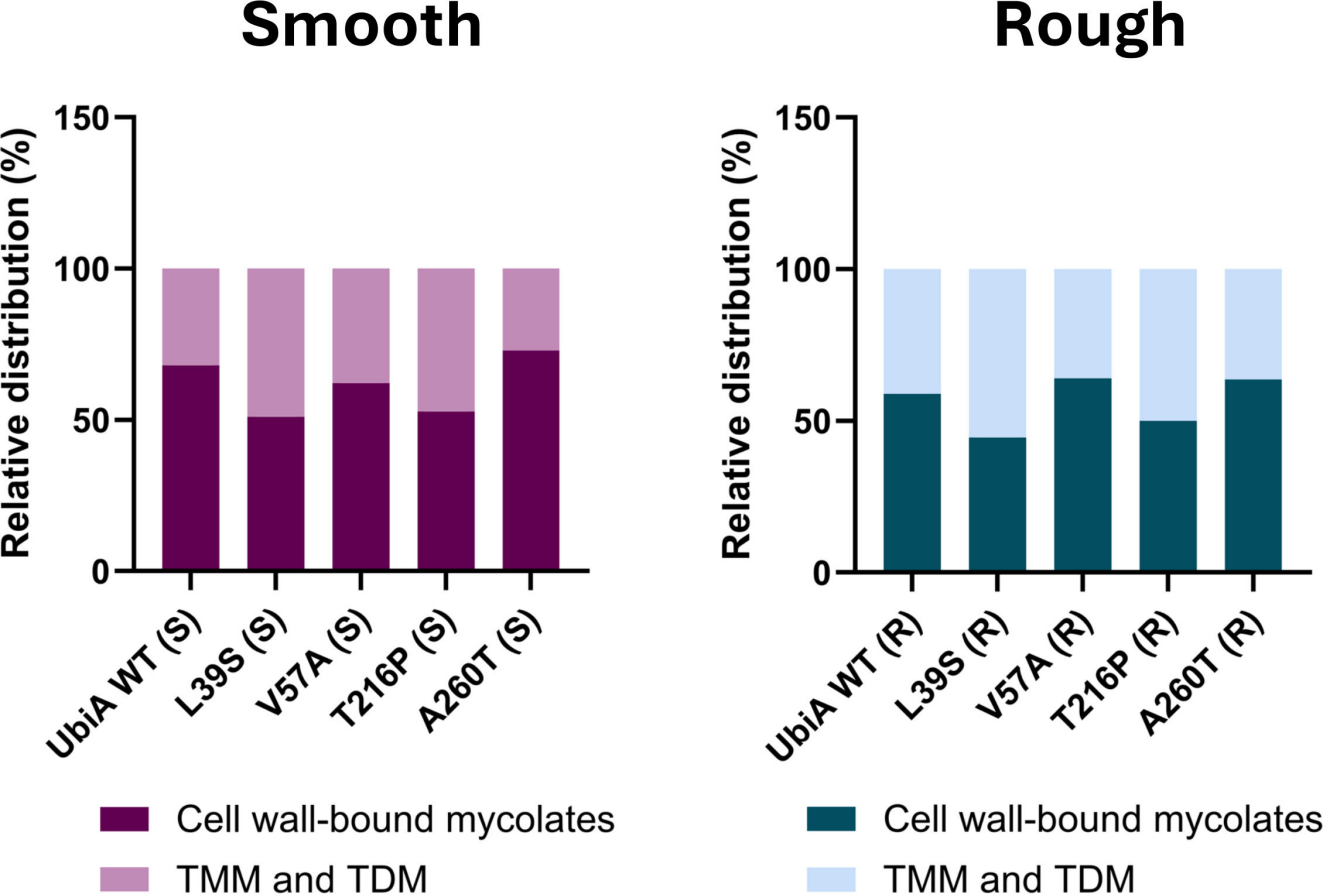
Impact of patient-derived *ubiA* mutations on the distribution of mycolic acids between the inner and outer leaflets of the outer membrane of *M. abscessus*. Smooth and rough WT and *ubiA* mutant strains were radiolabeled with [1,2-^14^C]acetate, and the amount of radioactivity incorporated into TMM + TDM and cell wall-bound mycolates determined by thin-layer chromatography as described under Materials and Methods. The graph shows the relative distribution (in percentages) of mycolic acids between TMM + TDM and cell wall-bound mycolates in each strain. The results of one labeling experiment are shown.

Both the terminal *t*-Ara*f* and the penultimate 2-Ara*f* of AG serve as esterification sites for cell wall-bound mycolates which make up the bulk of the inner leaflet of the outer membrane (22) (Fig. 1A). Mycolic acids also esterify non-covalently bound lipids of the outer membrane such as trehalose monomycolates (TMMs) and trehalose dimycolates (TDMs) (Fig. 1A). Since the relative percentages of *t*-Ara*f* and 2-Ara*f* of some S and R mutant AGs were altered (Table 3) and because it has previously been shown that the loss of mycolyl attachment sites on AG channels mycolates to TMM and TDM (33, 34), we set out to analyze the cell wall-bound mycolate and mycolylated lipid content of all R and S *ubiA* mutants. The distribution of *de novo* synthesized mycolates between TMM + TDM and the cell wall core (AG-bound mycolates) was analyzed upon metabolic labeling of the cells with [1,2-^14^C]acetate and quantification of the radioactivity incorporated into both populations of mycolates (see Materials and Methods for details and Fig. S3). Compared to their respective WT controls, the UbiA^L39S^ and UbiA^T216P^ R and S mutants displayed a relative ~10% to 17% increase in the TDM + TMM content, indicative of a reduced mycolylation of the cell wall core and a channeling of excess mycolates into outer membrane trehalose-based lipids (Fig. 3; Fig. S3). The results of one labeling experiment are shown, and percentages may slightly vary from batch to batch.

### Impact of patient-derived *ubiA* mutations on the growth and cell surface properties of *M. abscessus*

Given the more or less pronounced quantitative and/or qualitative changes in LAM, AG, and mycolic acid-containing lipids undergone by the UbiA mutants, we next investigated the impact such significant changes in the cell envelope might have on the growth rate, colony morphotype, surface hydrophobicity, biofilm formation, and ability of *M. abscessus* to grow as serpentine cords. All of these properties could indeed affect the interactions of the bacterium with the host in the course of infection.

In standard liquid medium (7H9-ADC-0.05% Tween 80), UbiA^T216P^ (S) was the only S morphotype mutant to present a slight growth retardation compared to the control strain. This phenotype was exacerbated by increasing the Tween 80 concentration to 0.5% (Fig. S4A). This finding suggests important alterations in the cell envelope permeability of UbiA^T216P^ (S), consistent with the increased susceptibility of this mutant to a number of antibiotics (Table 1). In synthetic cystic fibrosis medium (SCFM), which we found to be a better mimic of the composition of the CF airway (35), all S mutants grew comparably to UbiA^WT^ (S). With regard to the R morphotype strains, all mutants except UbiA^T216P^ (R) grew slightly slower than the control in 7H9-ADC-0.5% Tween 80, with UbiA^L39S^ (R) consistently being the most delayed. All R mutants, however, grew similarly to the UbiA^WT^ (R) control in SCFM with the exception of UbiA^T216P^ (R), whose delayed growth could tentatively be explained by the presence of 0.5% tyloxapol in the medium (required in the case of R strains for proper bacterial dispersion and OD_600_ measurements) (Fig. S4B).

Regarding colony morphology, while no obvious changes were observed with the R morphotype mutants, UbiA^A260T^ (S) was the only S mutant to present a glossier appearance compared to the control, UbiA^WT^ (S) (Fig. S5A). This observation correlated with the fact that UbiA^A260T^ (S) was also the only (S or R) mutant to present a more hydrophobic surface compared to the control as assessed by Congo red binding (Fig. S5B).

**Fig 4:**
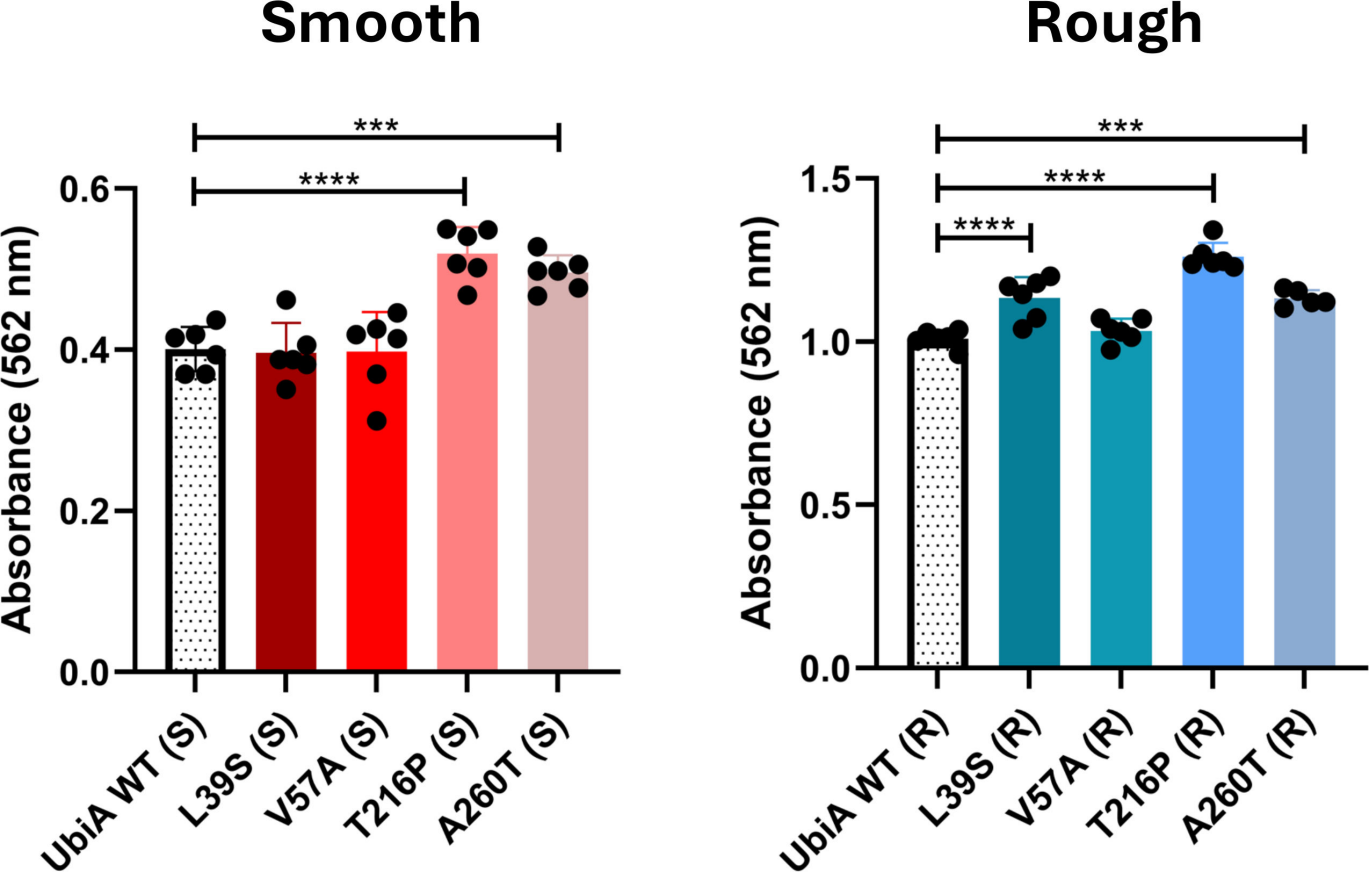
Patient-derived *ubiA* mutations enhance the biofilm-forming capacity of rough and smooth morphotype *M. abscessus*. Biofilm formation in SCFM by smooth and rough *ubiA* mutant strains was quantified after 5 days of incubation by crystal violet staining. Shown are averages ± SDs for six wells per strain, and the results are representative of two and three independent experiments for the S and R mutants, respectively. A one-way analysis of variance with Dunnett’s multiple comparison test against UbiA^WT^ (R/S) was performed. ***P < 0.001, ****P < 0.0001.

Finally, while UbiA mutations had no noticeable effects on the ability of R mutants to form serpentine cords (Fig. S6), more significant alterations were observed at the level of biofilm formation. In both the S and the R morphotypes, the UbiA^T216P^ and UbiA^A260T^ mutants consistently formed more biofilms in SCFM than their respective UbiA^WT^ controls (Fig. 4). Although not observed in the S morphotype, the L39S mutation also increased the biofilm-forming capacity of UbiA^L39S^ (R) (Fig. 4).

Overall, while these *in vitro* assays again revealed morphotype-specific phenotypes, a common trait that emerged was the association of several UbiA mutations with increased biofilm formation.

### *ubiA* mutants induce a greater pro-inflammatory response.

Results so far indicated that the mutants with the greatest number of divergent phenotypes compared to UbiA^WT^ (S/R) were UbiA^L39S^, UbiA^T216P^, and UbiA^A260T^. For these reasons, we proceeded with these mutants for further assessment of the impact of patient-derived *ubiA* mutations on *M. abscessus* S and R interactions with human innate immune cells.

**Fig 5:**
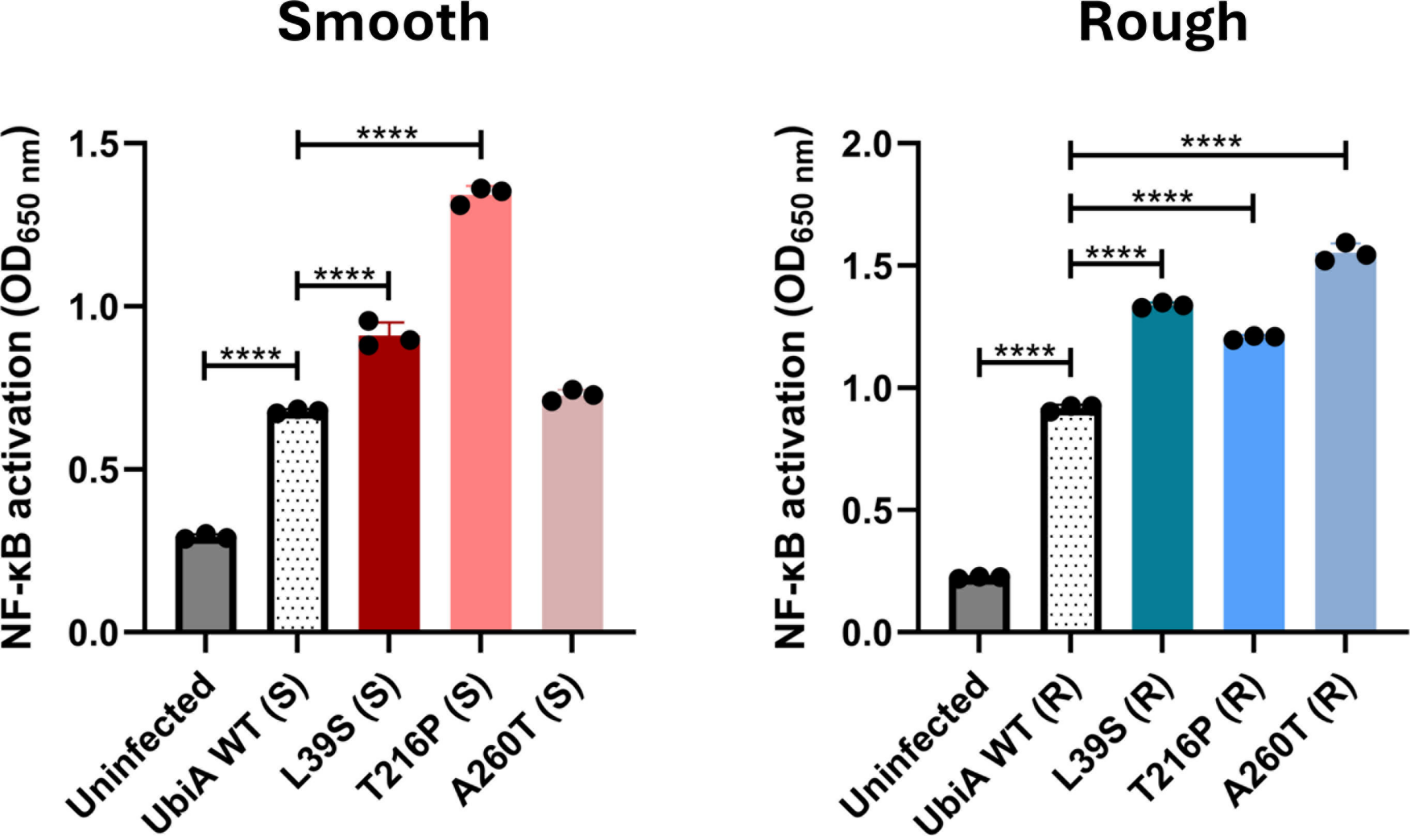
NF-κB activation in HEK-TLR2 cells by smooth and rough *M. abscessus* control and *ubiA* mutant strains. Activation of the TLR2-NF-kB axis was assessed using the reporter cell line HEK-Blue-hTLR2. Shown are averages ± SDs of absorbances measured at 650 nm for triplicate wells for each strain, and the results are representative of two independent experiments. A one-way analysis of variance with Dunnett’s multiple comparison test against UbiA^WT^ (R/S) was performed. ****P < 0.0001.

The induction of protective cytokines by murine and human macrophages infected with *M. abscessus* has been shown to depend in part on TLR2 signaling (13, 14, 36, 37). *M. abscessus* indeed displays various immunogenic molecules on its cell surface that signal through the TLR2-NF-κB axis, including phosphatidylinositol mannosides, lipoproteins, lipomannan (LM), and LAM (13, 14, 38). Given the alterations in cell surface properties displayed by some UbiA mutants (see the previous section), we first compared the ability of the different strains to induce an innate immune response using the HEK-Blue-hTLR2 reporter cell line. Interestingly, UbiA^L39S^ (S/R), UbiA^T216P^ (S/R), and UbiA^A260T^ (R) all activated TLR2 significantly more than their respective control (Fig. 5). Given that LAM purified from the same rough UbiA^L39S^, UbiA^T216P^, and UbiA^A260T^ mutants tended, on the contrary, to be either slightly weaker agonists (UbiA^T216P^ and UbiA^A260T^ LAMs) or as potent an agonist (UbiA^L39S^ LAM) as LAM purified from UbiA^WT^ (R) (Fig. S7), we conclude from this experiment that the modified LAM exposed by the mutants at their cell surface is not the primary driver of their increased TLR2 agonist activity. Instead, it is likely that other more potent TLR2 agonists (e.g., lipoproteins and LM) (14, 38), whose exposure at the cell surface and accessibility to TLR2 may have been indirectly affected by the *ubiA* mutations, account for this effect.

**Fig 6:**
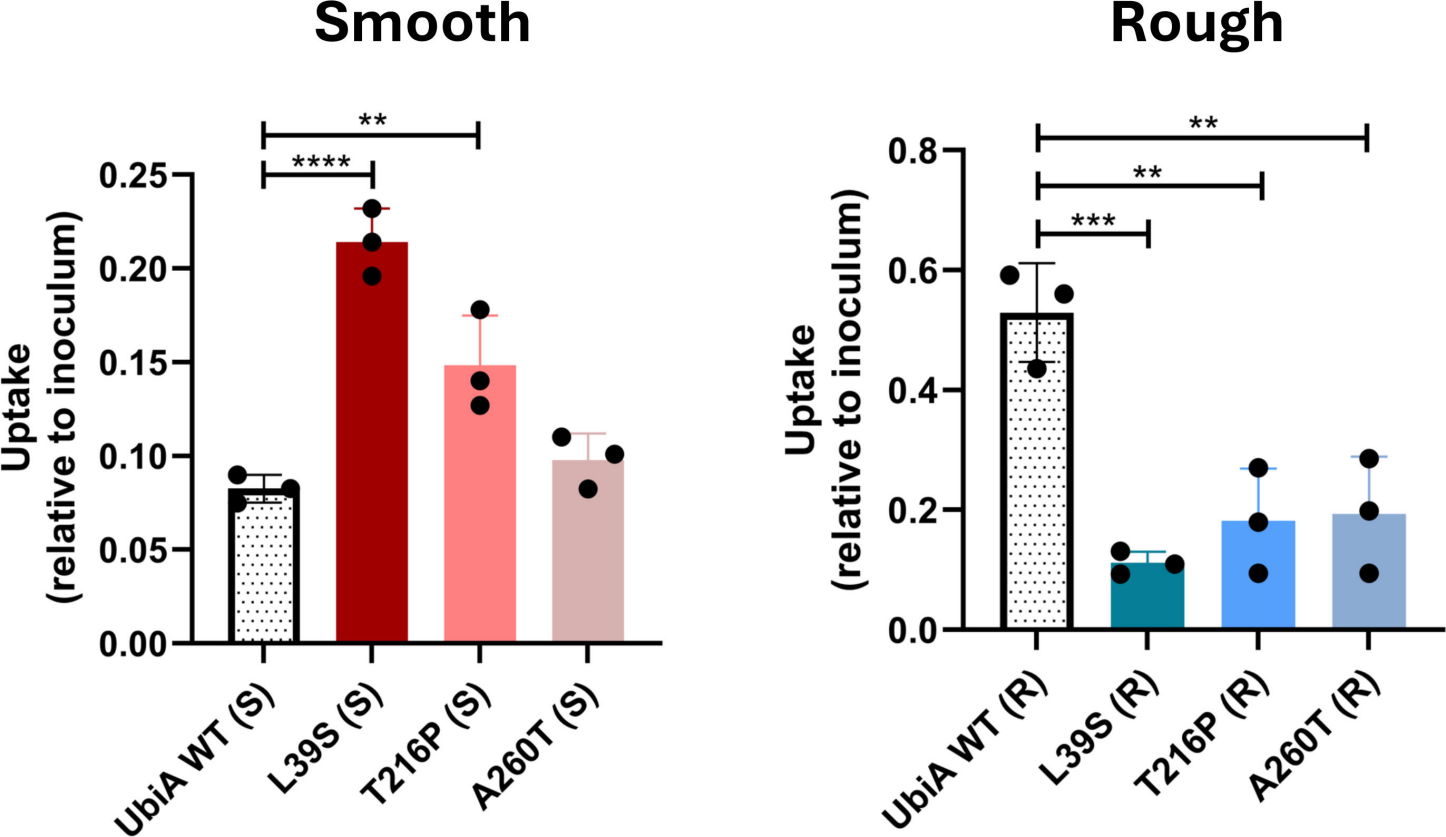
Effect of patient-derived *ubiA* mutations on the uptake of smooth and rough *M. abscessus* by monocyte-derived THP-1 macrophages. THP-1 macrophages were infected at a multiplicity of infection of 1. The uptake of bacilli at 2 h post-infection in phorbol 12-myristate 13-acetate-differentiated THP-1 macrophages is presented as a ratio of bacterial counts (CFU/mL) at 2 h post-infection relative to the inoculum for smooth and rough mutants. Shown are averages ± SDs of triplicate wells for each strain, and the results are representative of two and three independent assays for S and R mutants, respectively. A one-way analysis of variance with Dunnett’s multiple comparison test against UbiA^WT^ (R/S) was performed. **P < 0.01, ***P < 0.001, ****P < 0.0001.

To test the hypothesis that mutations in *ubiA* in R and S *M. abscessus* could indirectly induce a more pro-inflammatory response, we next used THP-1 monocytes differentiated into a macrophage subtype with phorbol 12-myristate 13-acetate to assess and compare the ability of the different *ubiA* mutants to infect, replicate, and induce innate immune responses. Strikingly, the uptake of all three R mutants by THP-1 cells was significantly reduced compared to the control, UbiA^WT^ (R) (Fig. 6). In contrast, uptake of S mutants was increased compared to the control, with the exception of UbiA^A260T^ (S), for which uptake was comparable to UbiA^WT^ (S) (Fig. 6). Once inside the cells, UbiA^L39S^ (S) and UbiA^T216P^ (S) replicated similarly to the control over 72 h, whereas UbiA^A260T^ (S) apparently failed to replicate during the same time period (Fig. S8A). No significant differences in intracellular survival/replication were observed between R strains (Fig. S8A).

**Fig 7:**
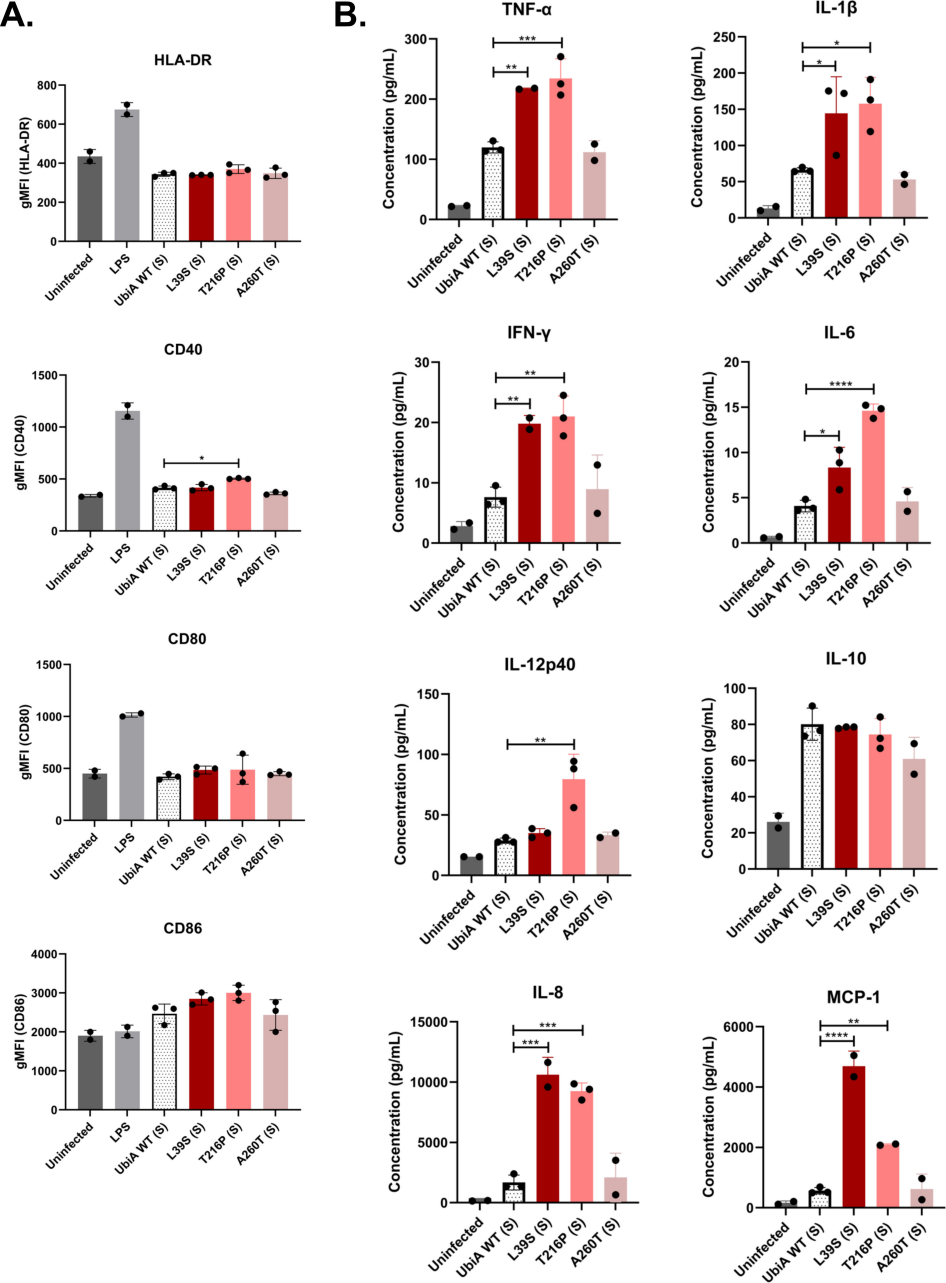
Immune activation and chemokine/cytokine secretion induced by the smooth control and *ubiA* mutant strains in monocyte-derived THP-1 macrophages. THP-1 cells were infected at a multiplicity of infection of 1. At 48 h post-infection, the (**A**) expression of activation markers on the surface of the cells and (**B**) secretion of cytokines and chemokines were measured by flow cytometry and an immunoplex assay, respectively. Shown are averages ± SDs of triplicate wells for each strain, and the results are representative of two independent assays. A one-way analysis of variance with Dunnett’s multiple comparison test against UbiA^WT^ (S) was performed. *P < 0.05, **P < 0.01, ***P < 0.001, ****P < 0.0001.

**Fig 8:**
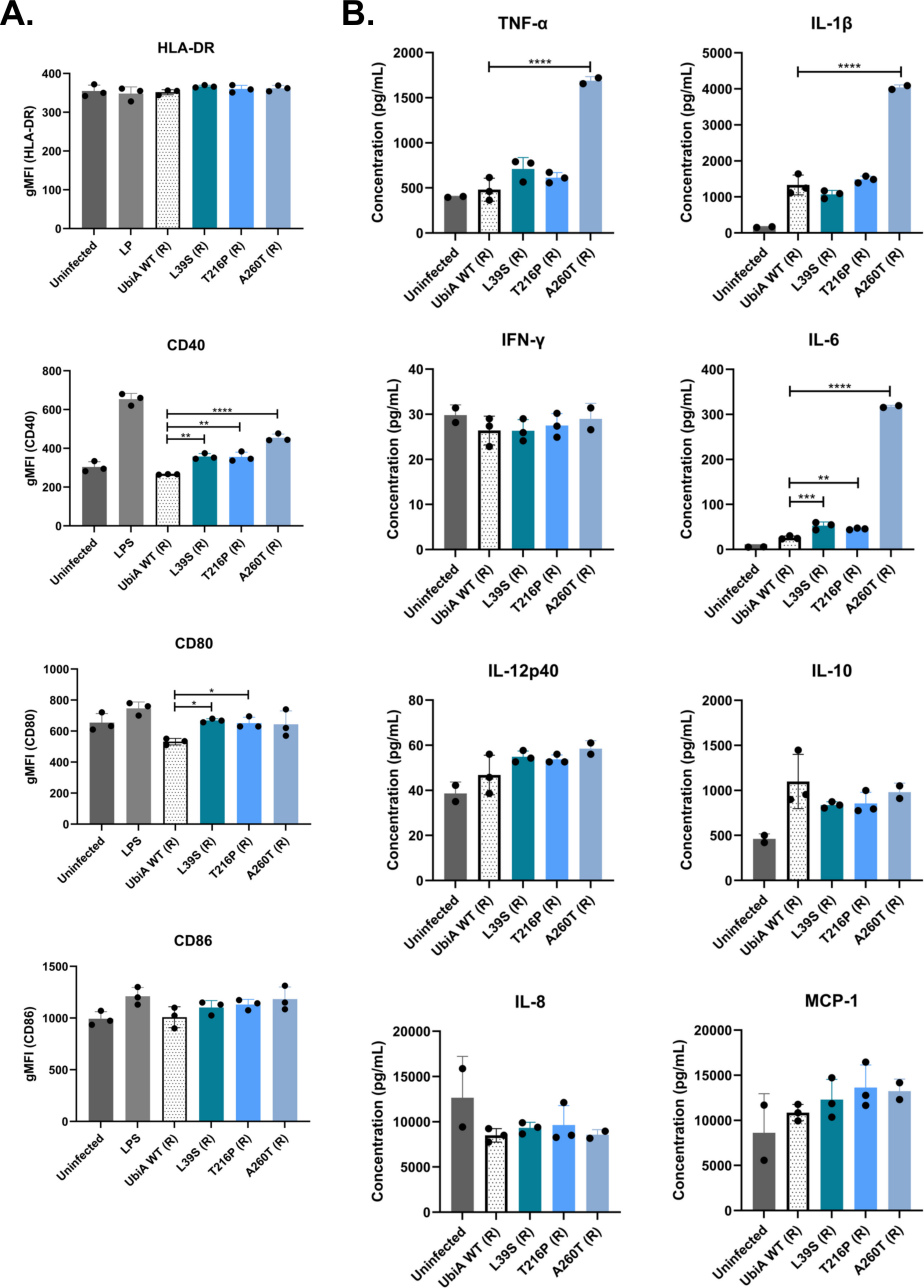
Immune activation and chemokine/cytokine secretion induced by the rough control and *ubiA* mutant strains in monocyte-derived THP-1 macrophages. THP-1 cells were infected at a multiplicity of infection of 1. At 48 h post-infection, the (**A**) expression of activation markers on the surface of THP-1 macrophages and (**B**) secretion of cytokines and chemokines were measured as described in Fig. 7. Shown are averages ± SDs of triplicate wells for each strain, and the results are representative of three independent assays. A one-way analysis of variance with Dunnett’s multiple comparison test against UbiA^WT^ (R) was performed. *P < 0.05, **P < 0.01, ***P < 0.001, ****P < 0.0001.

The inflammatory profile of infected THP-1 cells was assessed via the expression of activation markers and cytokine and chemokine secretion. In the S morphotype, no significant differences were noted between strains at the level of the activation markers HLA-DR, CD40, CD80, and CD86, with the exception of UbiA^T216P^ (S), which induced slightly more expression of CD40 (Fig. 7A). However, it was apparent from the cytokine and chemokine profiles that the UbiA^L39S^ (S) and UbiA^T216P^ (S) mutants were more inflammatory than UbiA^WT^ (S) and UbiA^A260T^ (S) based on significantly increased secretion of tumor necrosis factor alpha (TNF-α), interleukin-1β (IL-1β), interferon gamma, IL-6, IL-12p40, IL-8, and MCP-1. As suggested by the enhanced stimulation of TLR2 by the same two mutants (Fig. 5), UbiA^T216P^ (S) was the most inflammatory strain followed by UbiA^L39S^ (S) (Fig. 7B). In the R morphotype, significantly more expression of CD40 was observed in THP-1 cells infected with all three mutants. In addition, significantly more expression of CD80 was seen in UbiA^L39S^ (R)- and UbiA^T216P^ (R)-infected cells (Fig. 8A). Unlike the situation with S morphotype strains, but again in line with TLR2 stimulation results (Fig. 5), UbiA^A260T^ (R) was the most pro-inflammatory mutant, with significant increases in TNF-α, IL-1β, and IL-6 secretion in cells infected with this mutant compared to those infected with UbiA^WT^ (R). UbiA^L39S^ (R)- and UbiA^T216P^ (R)-infected cells also secreted significantly more IL-6 (Fig. 8B). Thus, overall, *ubiA* mutations in both morphotypes tended to augment the pro-inflammatory phenotype of *M. abscessus*.

### Interaction of *ubiA* mutants with A549 lung epithelial cells

**Fig 9:**
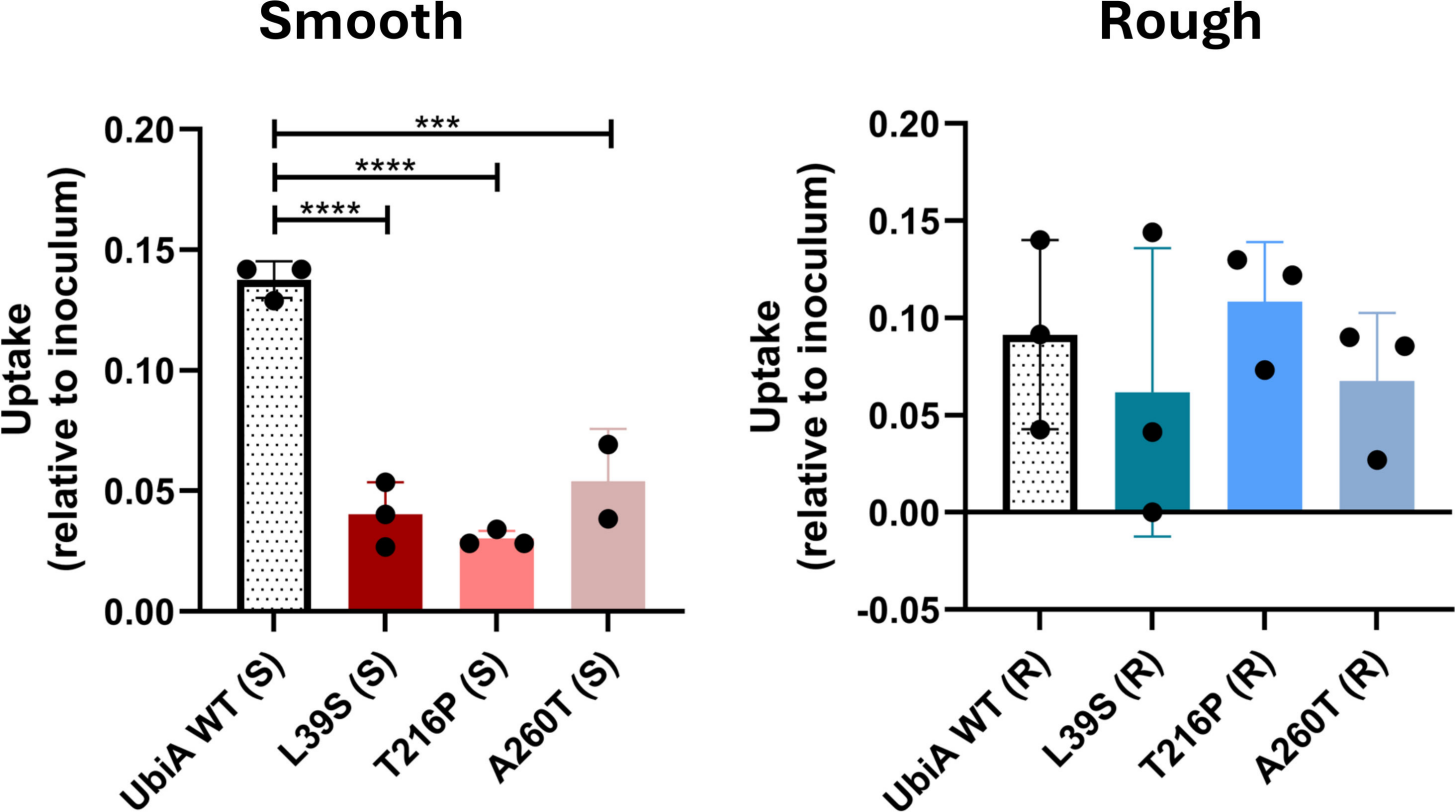
Effect of patient-derived *ubiA* mutations on the uptake of smooth and rough *M. abscessus* by A549 epithelial cells. A549 cells were infected at a multiplicity of infection of 1. The uptake of bacilli at 2 h post-infection is presented as a ratio of bacterial counts (CFU/mL) at 2 h relative to the inoculum for S and R mutants in A549 epithelial cells. Shown are averages ± SDs of triplicate wells for each strain, and the results are representative of three independent assays using different bacterial cultures. A one-way analysis of variance with Dunnett’s multiple comparison test against UbiA^WT^ (R/S) was performed. ***P < 0.001, ****P < 0.0001.

Macrophages are the primary population of cells that are infected by and initiate an immune response against mycobacteria, but lung epithelial cells are also engaged in these activities (39, 40). Human A549 alveolar epithelial cells were used to model this interaction. Interestingly, the uptake of *ubiA* mutants into A549 cells was opposite of what was observed in THP-1 cells. Uptake was decreased for all S morphotype mutants relative to UbiA^WT^ (S), while uptake was unchanged for the R morphotype mutants (Fig. 9). Once inside the cells, the S and R mutants persisted similarly to their respective control over 48 h (Fig. S8B). One of the primary cytokines secreted by A549 cells in response to *M. abscessus* infection is IL-8^38^. An enzyme-linked immunosorbent assay (ELISA) assay revealed no changes in IL-8 secretion for any of the S and R strains (Fig. S9).

## DISCUSSION

The *ubiA* gene was found to be mutated at a higher rate than expected by chance in *M. abscessus* isolates recovered from long-term infected patients, and it was postulated that mutations in *ubiA* may facilitate the adaptation of *M. abscessus* to the human lung (5). Due to the essentiality of *ubiA* for the synthesis of AG and LAM and the potential implication of an altered cell envelope on host–pathogen interactions, the functional consequence of a subset of clinically relevant *ubiA* mutations was characterized. The studies herein show that four patient-derived *ubiA* mutations, despite exerting their own unique combination of phenotypes, generally altered the biosynthesis of LAM and AG; affected the distribution of mycolic acids between the inner and outer leaflets of the outer membrane; increased biofilm formation; enhanced or reduced bacterial uptake by innate immune cells, depending on morphotype; and rendered the mutants more pro-inflammatory.

Unlike the arabinan domain of LAM, which is dispensable for *M. abscessus* viability (18), that of AG is essential to mycobacteria for its primary role in cell envelope integrity. With a finite supply of DPA available in each *ubiA* mutant, prioritizing the synthesis of AG over LAM is a logical choice. This biological decision was reflected in the content and structure of these glycans, wherein the arabinan of LAM not only had a more altered structure than that of AG but also LAM abundance also significantly decreased in some *ubiA* mutants. In both LAM and AG, changes in the structure of the arabinan domain were accompanied by alterations in the mannan and galactan domains, respectively. It is possible that these subsequent alterations are compensating mechanisms aimed at restoring some structural integrity in the cell envelope. Alternatively, the NS SNPs may destabilize the UbiA protein, leading to changes in the UbiA interactome with downstream consequences on the activity of other AG and LAM biosynthetic enzymes. UbiA has indeed been reported in *Corynebacterium glutamicum* to physically interact with a number of enzymes involved in the biosynthesis of these two glycans (41), and similar interactions are expected in mycobacteria. In support of multiple enzymes being perturbed in the UbiA mutants, both the R and the S strains displayed significant changes in the relative proportion of their Ara_4_, Ara_5_, and Ara_6_ arabinan termini and their degree of substitution by acetyl and succinyl groups that cannot be explained by the activity of UbiA alone. Moreover, the more pronounced impact of some mutations (e.g., L39S) on the structure of LAM in the S compared to the R morphotype and the opposite impacts of some mutations, depending on morphotype (e.g., branching of the mannan domain of LAM), may tentatively be interpreted as UbiA-containing protein networks being affected to different degrees, depending on the cell envelope environment. The notion that destabilizing mutations in critical LAM biosynthetic enzymes may alter the activity of other LAM-related enzymes has precedence in our recent analysis of *M. abscessus* mutants harboring missense mutations in the arabinosyltransferase EmbC (18).

The *ubiA* mutations were hypothesized to result from the evolutionary pressure for *M. abscessus* to adapt to the human lung (5). Using THP-1 monocyte-derived macrophages and A549 alveolar lung epithelial cells to model the innate immune cells *M. abscessus* would encounter in the human lung, we found that *ubiA* mutants exhibited disparate patterns in uptake that were both dependent on the morphotype and the cell type. In THP-1 cells, uptake was decreased for all R morphotype strains, whereas there was a slight increase in uptake for UbiA^L39S^ (S) and UbiA^T216P^ (S). In A549 cells, uptake was decreased for all S morphotype mutants, while there was no significant change for the R morphotype strains. The different uptake pattern of *ubiA* mutants in THP-1 monocyte-derived macrophages vs A549 epithelial cells most likely reflects the different internalization pathways used by *M. abscessus* to enter these cells. It has been shown that A549 epithelial cells internalize both pathogenic and non-pathogenic mycobacteria (*M. tuberculosis* and *M. smegmatis*, respectively) through macropinocytosis, which is not receptor mediated (42, 43). *M. abscessus* is likely internalized by A549 cells through a similar mechanism. In contrast, internalization of bacteria into macrophages is driven by receptor-mediated phagocytosis. The receptors responsible for *M. abscessus* uptake are not as well defined as with *M. tuberculosis*, but there is evidence that *M. abscessus* interacts with CD81, CD43, mannose receptor, complement receptor 3, and potentially other receptors that interact with laminarin (with the exception of dectin-1) (44, 45). Overall, a decrease in uptake by innate immune cells is a clear evasion strategy by *M. abscessus* to avoid internalization, which, coupled with the ability of these mutants to increase biofilm formation, may represent an efficient way for the bacterium to establish an extracellular, drug-tolerant, and persistent infection in the lung. While mutations in other genes than *ubiA* (e.g., GLP biosynthetic genes, *embC*, and *phoR*) may result in similar changes in *M. abscessus* extracellular growth and interactions with innate immune cells, the fact that *ubiA* was identified as one of the top four loci accumulating NS SNPs in the course of infection clearly points to *ubiA* mutations as one of the preferred mechanisms of adaptation of *M. abscessus* to lung infection (5).

Another striking feature of patient-derived UbiA mutants was the significant trend by both morphotypes to induce a more pro-inflammatory response in HEK-Blue-hTLR2 and THP-1 cells. The benefit of a stronger pro-inflammatory response is that it shifts the delicate balance of the inflammatory response toward the pathological side, which can result in ineffective bacterial clearance as seen in *Pseudomonas aeruginosa* pulmonary infections (46, 47). In the case of UbiA^A260T^, this mutation was known to have occurred in a rough clinical isolate (the morphotype was not recorded for the remaining *ubiA* mutants) (5). That this mutation would preferentially occur in a clinical isolate only after it underwent a switch from S to R is supported by our findings that while UbiA^A260T^ (R) is significantly more pro-inflammatory than UbiA^WT^ (R), the corresponding UbiA^A260T^ (S) mutant was not only unchanged in its inflammatory profile from UbiA^WT^ (S) but also attenuated for replication in macrophages. The same might be said of the T216P mutation, which led to important cell envelope permeability defects in *M. abscessus* S (but not R), resulting in enhanced susceptibility to some antibiotics. It thus appears that certain UbiA mutations may only be beneficial in one morphotype.

Interestingly, and more strikingly so in the R morphotype, decreased LAM content appeared to be strongly associated with the induction of pro-inflammatory responses, increased biofilm formation, and reduced uptake by THP-1 cells. While a possible direct contributor to these phenotypes, LAM is unlikely to be the sole factor involved. Indeed, as recently illustrated in the case of EmbC mutants of *M. abscessus* (18), it is to be expected that the profound and pleiotropic changes directly or indirectly caused by *ubiA* mutations on the cell surface properties of the bacilli will significantly impact their aggregative properties and interactions with innate immune cells.

Mounting evidence indicates that rearrangements in the cell surface composition of *M. abscessus* leading to alterations in the biosynthesis of glycolipids and (lipo)polysaccharides play an important role in infection by directly and indirectly modulating the ability of the bacterium to form biofilms and cords and to interact with innate immune cells. R variants producing little to no GPLs are characterized by the ability to withstand phagocytosis, extracellular growth, and a hyper-pro-inflammatory TLR-2-dependent phenotype (9, 11–15). Patient-derived mutations in EmbC, the arabinosyltransferase responsible for elongating the arabinan domain of LAM, result in pleiotropic effects on the cording, sliding motility, biofilm-forming capacity, inflammatory properties, and intracellular replication of *M. abscessus* (18). Finally, other frequent adaptive mutations identified in the two-component system regulator PhoPR of *M. abscessus* clinical isolates lead to the upregulation of genes involved in intracellular survival and redox homeostasis, including a number of glycosyltransferases and glycosyl hydrolases of as yet unknown function (17). The mutations in *ubiA* described herein provide additional support to the idea that *M. abscessus* extensively relies on its cell envelope glycans to modulate its cell surface composition and properties and, thereby, to adapt its physiology and pathogenicity to the evolving pressures it experiences within the lung.

## MATERIALS AND METHODS

### UbiA mutant modeling and analysis

The predicted full-length sequence of the *M. abscessus* UbiA decaprenyl phosphate phosphoribosyl-transferase was downloaded from the AlphaFold database (AF-B1MEK9-F1) (48). The OPM program was used to position UbiA in the membrane bilayer and to visually classify the location of the mutated residues into one of four categories, namely, the bilayer, extracellular, cytoplasmic, and interfacial (either close to the extracellular or cytoplasmic regions) (49). Conserved Domain Database (CDD) was used to identify *M. abscessus* UbiA as a member of the UbiA family of prenyltransferase superfamily containing 95 members (CDD ID PRK12324) (50). The multiple sequence alignment of the family members obtained from CDD was used to calculate residue conservation using the Protein Variability Server (51). The binding sites of DP and PRPP were inferred from the structural alignment of UbiA from *M. tuberculosis* (PDB 8J8K and 8J8J, respectively) (52). The impact of mutations on protein stability was predicted using SDM (53) and FoldX (54). In addition to stability scores, three other structural properties were assessed for each mutation, including relative solvent accessibility (53), residue depth (55), and OSP density (56) (Table S1).

The impact of mutations on the binding of DP and PRPP to UbiA was predicted using PremPLI, a machine learning method to predict the change in ligand-binding affinity upon a point mutation (57). Finally, the Ohm program was used to study the impact of UbiA mutations on UbiA allostery (58). Given a protein structure and its ligand-binding sites, Ohm calculates the allosteric coupling intensity of all residues relative to the ligand-binding sites. The ligand-binding site is defined as a set of residues within the contact distance from the ligand atoms as defined by ChimeraX (59).

### Bacterial strains and culture media

*M. abscessus* ATCC 19977 S and R variants (60) and derived recombinant strains were grown under agitation at 37°C in Middlebrook 7H9 medium supplemented with 10% ADC and either 0.05% Tween 80 (S strains) or 0.5% Tween 80 (R strains) (7H9-ADC-Tween 80), in SCFM (61) with 0.05% tyloxapol (S strains) or 0.5% tyloxapol (R strains only), or on Middlebrook 7H11 agar supplemented with 10% oleic acid-albumin-dextrose-catalase (OADC). Zeocin, kanamycin, and hygromycin were added at 100 µg/mL, 200 µg/mL, and 2 mg/mL, respectively.

### Construction of *M. abscessus ubiA* mutants

Construction of the isogenic mutants began with creating merodiploids of *M. abscessus* ATCC 19977 (S/R) by transformation with an integrative plasmid (pMV306, harboring a kanamycin resistance cassette) expressing *ubiA^WT^* under the control of the *hsp60* promoter. Next, the ORBIT system (62) was used to delete the native *ubiA* gene and introduce an *hsp60* promoter downstream of the disrupted *ubiA* gene to restore expression of the *aftB* arabinosyltransferase gene (*MAB_0174*) required for AG and LAM biosynthesis (34, 63). Isogenic mutants expressing mutated variants of *ubiA* were generated by transforming the merodiploid R and S strains, *Mabs*Δ*ubiA*/pMV306-*ubiA^WT^*, with integrative pMV306H plasmids (harboring a hygromycin resistance cassette) expressing *ubiA^L39S^*, *ubiA^V57A^*, *ubiA^T216P^*, and *ubiA^A260T^* from the *hsp60* promoter followed by selection on 7H11-OADC plates containing hygromycin. Replacement of *ubiA*^WT^ with the mutated variants was confirmed by PCR and sequencing.

### Drug susceptibility testing

MIC values were determined in 7H9-ADC-Tween 80 broth in 96-well plates. *ubiA* mutants were diluted to a concentration of 10^5^ CFU/mL and incubated in the presence of serially diluted concentrations of compounds (256.0–0.0625 μg/mL) for 5 days (or 14 days for azithromycin to account for inducible macrolide resistance). MICs were determined using the resazurin microtiter plate assay method (64). By this method, the MIC was the first concentration where there was >90% loss of fluorescent signal relative to untreated wells. After the wells were incubated with resazurin overnight, fluorometric readings were acquired with the Synergy H1 microplate reader (BioTek) with excitation/emission at 530/590 nm. To calculate the relative fluorescent signal, (i) the mean background fluorescence was subtracted from each antibiotic-treated and untreated well; (ii) the relative fluorescence was calculated by dividing the fluorescent signal of each antibiotic-treated well with the mean fluorescence of the untreated wells; and (iii) multiplying the product by 100 to acquire the relative fluorescent signal (%). Each concentration was tested in duplicate.

### Purification and analysis of lipids, mycolic acids, LAM, and AG

Lipids, lipoglycans, and mAGP were extracted as previously described (18, 65–67). LAM was purified from a mixture of LAM, LM, and phosphatidylinositol mannosides by size exclusion chromatography using a Sephacryl S100HR (Sigma) column as described (18) and was analyzed by SDS-PAGE on commercial Novex 10%–20% tricine gels stained with periodic acid–Schiff reagent. The results presented in Fig. 2 are representative of two separate lipoglycan extractions and SDS-PAGE analyses. For glycosyl linkage analysis, pure LAM or mAGP samples (50 µg) were permethylated, hydrolyzed, reduced, and acetylated to yield the corresponding alditol acetates, which were then analyzed by gas chromatography-mass spectrometry (GC/MS) on a TSQ 8000 Evo triple-quad GC/MS instrument (Thermo Scientific) (66). Data analysis was performed using the Chromeleon Chromatography data system (Thermo Scientific). Oligoarabinosides released from LAM upon *Cellulomonas gelida* endoarabinanase digestion were analyzed by ultra-high performance liquid chromatography (UHPLC) on an Atlantis T3 column (Waters) using an Agilent 1290 Infinity II UHPLC system coupled to an Agilent 6546 quadrupole-time of flight mass spectrometry instrument (66). Data analysis was performed using the MassHunter data system (Agilent).

### Metabolic labeling

*ubiA* mutants grown to exponential phase (OD_600_ ~0.8) at 37°C in 7H9-ADC-Tween 80 were labeled with 0.5 µCi of [1,2-^14^C]acetate (52 mCi/mmol, PerkinElmer) and incubated for an additional 4 h before the cells were collected for total lipid and cell wall mycolate extraction and analysis. Radiolabeled lipids were analyzed for TMM and TDM content on silica gel 60-precoated TLC plates (Millipore Sigma) eluted with chloroform:methanol:water (20.0:4.0:0.5, by vol). Radiolabeled cell wall-bound mycolic acids were prepared from delipidated cells and derivatized to mycolic acid methyl esters as described previously (68). Radiolabeled mycolylated products were visualized using a Sapphire Biomolecular Imager and quantified using the AzureSpot analysis software (Azure Biosystems).

### Congo red binding

*M. abscessus* strains were tested for Congo red binding in tryptic soy agar as described (69). Briefly, *M. abscessus* grown to exponential phase were streaked on tryptic soy agar plates containing 100 µg/mL Congo red and incubated for 7 days at 37°C. The biomass was scraped off the plate, and cell-bound Congo red was extracted in acetone for 2 h at room temperature. The re-solubilized Congo red was read at OD_488_ on a Genesys 30 visible spectrophotometer (Thermo Scientific), and the measured values were normalized to the weight of the starting wet bacterial pellets.

### Cording assay

Assessment of cording was performed as described by De et al. (18). Briefly, *ubiA* control and mutant R strains were diluted to 10^4^ CFU/mL in tryptic soy broth and incubated in 35 mm glass bottom dishes (Ibidi). Growth and cords were observed for 3 days using an Olympus CKX41 microscope. Images were taken using an attached Olympus U-CMAD3 camera.

### Biofilm formation

Biofilm formation in SCFM was monitored by crystal violet staining as described by Belardinelli et al. (61). For each assay, six technical replicates were performed per strain.

### Determination of hTLR2-mediated NF-κB activity using HEK-Blue-hTLR2 reporter cells

HEK-Blue-hTLR2 cells (InvivoGen) were handled and cultured according to the manufacturer’s protocol. A total of 54,000 cells per well were seeded in 96-well plates in HEK-Blue detection medium and incubated at 37°C with 5% CO_2_. Frozen titered stocks of *ubiA* mutants and control strains were thawed and passed through a 26-gauge needle 40 times to achieve single-cell suspensions. *M. abscessus* strains were added to the wells at a multiplicity of infection (MOI) of 1. Alternatively, LAM purified from rough *ubiA* control and mutant strains, or LM and LAM purified from *Mycobacterium tuberculosis* H37Rv (BEI resources), were added to cells at a final concentration of 1 µg/mL. Plates were incubated at 37°C in a humidified incubator with 5% CO_2_, and hTLR2-mediated NF-κB activity was monitored spectrophotometrically at 650 nm with a Synergy H1 microplate reader (BioTek) after 16 h.

### Macrophage infections, flow cytometric analysis of activation markers, and multiplex immunoassay for cytokines and chemokines

THP-1 cell (ATCC TIB-202) infections, flow cytometric analysis of activation markers, and analysis of cytokines and chemokines followed earlier procedures (18).

### Infection of A549 lung alveolar type II epithelial cells

A549 human lung alveolar type II epithelial cells (ATCC CCL-185) were grown in 24-well plates to ~90% confluence in Dulbecco’s modified Eagle medium (Gibco) supplemented with 10% fetal bovine serum and 1% penicillin–streptomycin. Cells were infected as described previously (18) at an MOI of 1 using frozen titered stocks of *M. abscessus* passed through a 26-gauge needle 40 times to achieve single-cell suspensions. Supernatants collected 48 h post-infection were assessed for IL-8 production using the human IL-8 DuoSet ELISA kit (R&D Systems).

### Statistical analysis

Statistical tests were performed as indicated in the figure legends. Calculations were performed using GraphPad Prism version 10.0.0 for Windows (GraphPad Software).

## SUPPLEMENTAL MATERIAL

Supplemental material mbio.00322-25-s0001.pdf Tables S1 to S3 and Figures S1 to S9.

ASM does not own the copyrights to Supplemental Material that may be linked to, or accessed through, an article. The authors have granted ASM a non-exclusive, world-wide license to publish the Supplemental Material files. Please contact the corresponding author directly for reuse.
