# Supplementary material for: Cell envelope polysaccharide modifications alter the surface properties and interactions of Mycobacterium abscessus with innate immune cells in a morphotype-dependent manner
Source: mBio. 2025 Mar 14;16(4):e00322-25. doi: 10.1128/mbio.00322-25 (PMC11980365; doi:10.1128/mbio.00322-25)

Cell envelope polysaccharide modifications alter the surface properties and interactions of *Mycobacterium abscessus* with innate immune cells in a morphotype-dependent manner

Elena Lian[a], Juan M. Belardinelli[a], Kavita De[a], Arun Prasad Pandurangan[b], Shiva K. Angala[a], Zuzana Palčeková[a], Anna E. Grzegorzewicz[a], Josephine M. Bryant[c], Tom L. Blundell[b], Julian Parkhill[d], R. Andres Floto[e,f,g], William H. Wheat[a], Mary Jackson[a#]

Table of Contents

Table S1: Predicted impact of mutations on protein stability and their structural properties.

Table S2: Susceptibility to antibiotics of smooth and rough *ubiA* mutant strains in 7H9-ADC-Tween 80 (raw data associated with the resazurin assay results presented in Table 1).

Table S3: LC/MS analysis of the unmodified and covalently modified oligoarabinosides released from the LAM of smooth and rough *ubiA* mutants upon endoarabinanase digestion.

Figure S1: Predicted impact of the fourteen UbiA mutations on the binding affinity of DP and PRPP.

Figure S2: Allelic replacement at the *ubiA l*ocus of rough and smooth merodiploid *M. abscessus* ATCC 19977 strains.

Figure S3: Impact of patient-derived *ubiA* mutations on the distribution of mycolic acids between the inner and outer leaflets of the outer membrane of *M. abscessus* (autoradiograms).

Figure S4: Growth of smooth and rough *ubiA* mutant strains in 7H9-ADC-Tween 80 and SCFM at 37°C.

Figure S5: Impact of patient-derived *ubiA* mutations on the morphology and surface hydrophobicity of smooth and rough *M. abscessus*.

Figure S6: *ubiA* mutations do not alter the ability of rough *M. abscessus* to form cords.

Figure S7: NF-κB activation in HEK-TLR2 cells by purified LAM from rough *M. abscessus* control and *ubiA* mutant strains.

Figure S8: Intracellular replication of *ubiA* mutants in human monocyte-derived THP-1 macrophages and A549 epithelial cells.

Figure S9: IL-8 secretion induced by the control and *ubiA* mutant strains in human A549 epithelial cells.

<u>Table S1</u>. Predicted impact of mutations on protein stability and their structural properties. SDM and FoldX mutant stability prediction: SDM score are unitless whereas the FoldX score are given in kcal/mol. Positive and negative values correspond to stabilizing effects in SDM and FoldX, respectively, and vice versa. OSP: occluded surface packing. *Mutations studied experimentally.

Mutation	FoldX ΔΔG (kcal/mol)	SDM pseudo ΔΔG	Relative solvent accessibility (%)	Residue depth (Å)	Occluded Surface Packing (OSP)	OPM classification
A217V	-0.16	-0.84	29.80	3.63	0.39	Cytoplasm
D44G	0.24	-0.31	75.90	3.33	0.14	Extracellular
**V57A***	2.11	-0.87	1.00	5.63	0.60	Bilayer
**L39S***	0.89	-1.41	68.40	3.44	0.30	Bilayer
W177G	5.0	-1.28	27.80	4.41	0.41	Bilayer
V285A	0.26	0.84	63.20	3.52	0.34	Interface
A246V	1.17	-0.12	73.60	2.99	0.22	Extracellular
**T216P***	2.71	-1.84	75.80	3.43	0.29	Cytoplasm
D269G	1.62	-0.17	48.30	3.75	0.26	Cytoplasm
W249R	0.18	0.21	58.40	3.8	0.30	Bilayer
G247D	2.52	-2.5	98.80	3.36	0.18	Interface
L187F	1.65	-0.63	17.80	4.29	0.46	Bilayer
**A260T***	2.23	-2.05	31.60	3.74	0.50	Bilayer
F257S	7.23	-0.42	17.60	4.65	0.47	Bilayer

Table S2: Susceptibility to antibiotics of smooth and rough *ubiA* mutant strains in 7H9-ADC-Tween 80 (raw data associated with the resazurin assay results presented in Table 1).

The raw data are presented with cosmetic edits to indicate the orientation of the plates. All cosmetic edits are indicated in the legend.

Legend

Antibiotic	Abbreviation	Concentration range[1]
Rifampin	RIF	0.25-128 µg/mL
Rifabutin[2]	RFB	0.25-128 µg/mL
Vancomycin	VAN	0.25-128 µg/mL
Linezolid	LZD	0.25-128 µg/mL
Cefoxitin	FOX	0.25-128 µg/mL
Amikacin	AMK	0.25-128 µg/mL
Levofloxacin	LEV	0.25-128 µg/mL
Tigecycline	TGC	0.25-128 µg/mL
Ethambutol	EMB	0.5-256 µg/mL
Azithromycin[2]	AZT	0.5-256 µg/mL

[1]Concentrations were organized on the plate from high to low concentration, left to right.

[2]A different concentration range was used in a second experiment for a few smooth *Mabs ubiA* strains to confirm MIC results. In cases where a different concentration range was used, it was noted below the raw data.

Blue values = background wells (medium only)

Orange values = untreated wells (medium + bacteria, ~ 0 µg/mL)

Highlighted values = Background, untreated, and azithromycin-treated wells were stained with resazurin on day 13 and fluorometric readings were obtained on day 14.

UbiA^{WT} (S)

	29206	27071	27497	27110	26842	27072	26874	26913	26981	27198	27312	27902
RIF	29185	46383	35136	28716	25814	25257	26913	82395	110006	135176	146183	214291
	28419	45805	34616	28374	25213	24820	27451	99884	111743	113616	131984	177000
RFB	27346	26990	26165	26405	26523	26210	26434	27130	95981	119080	139148	158024
	27024	26929	27058	26460	26548	26534	26567	38507	107658	115080	129679	152771
VAN	26077	26004	24713	24958	24625	24419	24412	108291	117184	120002	123952	159959
	26884	25641	25348	25045	24805	24815	24669	103356	116264	123212	124697	172226
	133322	142631	148759	150264	147588	143930	146950	147664	148798	153057	152197	204074
	25079	27917	27066	26762	26570	26403	26604	26623	26553	26810	27159	28360
LZD	28471	28715	27032	26228	25589	26226	27203	41141	116539	109423	121761	176964
	24880	29069	27178	26057	24847	25584	27907	44124	111546	113338	132590	154944
FOX	28292	121470	69671	46972	35845	30543	36666	134242	128488	132300	150566	148383
	27800	119205	69913	45735	34698	31059	31437	132865	125915	129534	114892	153244
AMK	24917	30712	27457	26267	31277	148753	107649	116134	124891	124227	125424	143303
	25200	29417	27562	26642	25943	137529	101768	106506	113999	122846	122841	154984
	119379	158554	152933	153485	148404	146556	140110	138753	140937	127749	137557	207485
	29130	27756	27314	27299	26931	27026						
LEV	28058	27200	25708	25214	24175	24162	24635	23979	52448	113646	130917	250332
	27505	26790	25276	24732	24172	23740	23480	27865	73770	106768	126762	150551
TGC	28065	53708	41338	33619	29421	27328	26130	35673	92810	112998	110726	155156
	28702	53878	42379	35051	27494	27349	26358	33950	104686	119044	87105	157449
							146654	146326	145505	153485	150747	199606
	30495	29170	28540	27385	27786	28421	37275	37033	35429	36495	35979	32721
EMB	29828	33209	29763	28810	43912	120988	122840	117034	106918	106586	130039	178976
	29293	33131	29399	29312	39860	113429	117020	109900	106126	96369	108345	141249
AZT	38098	36908	106796	159773	206537	251641	232943	267713	266181	261774	282512	223151
	52598	42268	108817	137485	159287	241522	238754	251087	240993	268788	281673	308556
	220300	280199	297794	313564	303641	305542	160602	211733	208309	182577	188617	256300

UbiA^{L39S} (S)

	29221	27841	27451	26846	27109	27101	27207	26989	26357	27112	27982	28092
RIF	27956	47001	34868	28328	25244	24392	23930	27260	82397	127410	128182	242564
	27879	46674	35229	28339	24950	24013	23317	25860	67538	121945	146024	192252
RFB	27821	20290	22093	22523	22846	23299	23441	24010	24285	25277	58896	185632
	27592	19997	21865	22776	22352	23836	23611	24686	24801	25459	50641	184910
VAN	27979	25853	24997	24509	24242	24299	24141	24583	144573	136942	156836	217101
	28813	26288	25514	25516	24852	24836	24823	24930	137261	121510	145361	201219
	199299	188695	146304	159071	155983	147742	141586	156271	194557	146737	152750	191756
	29324	28402	27529	26999	26812	25788	26456	26197	26466	26589	27081	28227
LZD	28549	29560	26644	26158	24806	24759	25271	28571	85686	113305	111019	187780
	28222	29762	27030	25852	24851	24824	24704	27899	89844	119366	116146	146587
FOX	28409	123965	70935	46163	35611	30301	27355	116753	141926	130047	125503	152755
	28205	118782	70281	45978	34253	29961	39766	119043	135698	115789	138003	180835
AMK	28525	31900	28329	26433	25521	64140	109650	107790	130619	135779	148542	201088
	29009	31534	27572	26358	26160	150911	112403	116755	129077	134455	146088	211061
	201349	160233	157783	151992	150265	142894	154051	147895	179228	183113	185273	204608
	30256	29603	28678	28422	28468	28439						
LEV	29877	28424	26079	25765	24944	24949	24751	25653	45426	131691	144191	23058
	29607	27678	26360	25238	24736	24540	24339	24626	34572	123316	139809	23408
TGC	30118	57458	43750	35500	30559	28024	27744	35084	71044	130332	130312	23401
	30083	57837	44463	36025	30687	28438	27825	33386	68514	130622	125954	23832
							145584	146007	146532	142278	142496	201391
	28154	27721	27798	27829	27148	26197	26230	26380	26819	26717	27014	27111
	37432	38113	37470	37742	36674	36024	161040	154805	148058	182186	148778	282841
EMB	29091	34922	30545	28986	31123	87783	130755	154582	194333	182710	206438	218597
	29260	35553	33173	29493	31305	87774	139340	159125	178084	192693	196734	221167
AZT	37467	34445	114392	114625	104842	123958	238332	247708	272660	282857	249083	322157
	37637	34255	114434	113486	124549	140892	250119	275214	276903	278683	284653	298404
	263160	222962	178931	195934	179874	196850	195169	194567	200016	178296	214028	270057

UbiA^{V57A} (S)

	29431	28233	27167	27491	27299	27062	27047	27606	27459	27299	27763	28842
RIF	28918	51461	38448	30598	27352	26535	25370	28701	100846	145718	155305	181581
	27793	48009	36055	30280	26178	26904	40927	30296	102923	136288	141167	148594
RFB	27732	18437	22636	23477	23403	26516	35160	31796	29889	25466	122544	156175
	27477	20933	25261	21454	26083	22989	24540	24664	22807	25619	118935	151396
VAN	26720	27156	25402	25078	24821	23241	23302	22511	125132	135074	146517	159856
	27932	25954	25271	25032	24837	25054	25290	131300	126587	134294	145237	193375
	236507	157938	189394	183817	190548	192989	191206	205339	206683	195756	225409	204454
	29196	27861	27184	27563	27067	27344	27014	27056	27289	26939	27347	27943
LZD	28187	29987	28120	26663	25260	25474	26815	31728	118950	118745	119526	153203
	28426	30044	27457	25790	25219	24954	25556	30981	93201	103133	114043	144896
FOX	28883	124484	70474	47045	36040	30178	27580	150879	146552	136987	136853	151795
	28904	129643	69822	46605	35299	30116	27629	161417	130442	148301	135759	162366
AMK	28944	31145	27942	26621	25922	146413	93458	136674	122310	133388	131047	192772
	28828	31130	27140	26676	46029	142996	99417	109110	116104	143471	132984	189759
	256128	223905	148537	175476	151609	157434	152747	155965	199042	200285	200613	228759
	29818	28629	28380	27714	27825	27919						
LEV	29151	27409	26054	25355	24771	24425	25165	24924	30718	112507	146434	140081
	28950	27071	25464	24830	24021	23694	24139	24350	87128	118991	136555	139853
TGC	28488	61875	44881	37750	31039	28138	27382	28152	54789	124053	127547	147022
	29928	60989	44845	35864	31434	27950	26834	30190	77250	124186	128633	156637
							189276	189589	199884	193040	228716	205538
	29400	29259	29040	28481	29003	28396	39321	41266	38745	36497	38545	35528
EMB	30040	33132	29676	28323	32672	104790	148915	132397	123994	95735	151562	137430
	29940	32991	29561	28251	32151	94577	119021	121400	116439	114692	119470	145544
AZT	40997	34163	108296	105196	114540	115334	131848	193340	242479	167362	153607	280824
	46699	33944	111299	114706	107951	110443	259433	200813	266355	164134	300517	309036
	245838	275278	271548	294150	292688	298535	230610	236323	221189	216694	176971	218635

Azithromycin concentration range: 1-512 μg/mL

UbiA^{T216P} (S)

	25655	27361	27150	26919	26932	27083	26446	26822	26674	27063	27271	26903
RIF	28012	46910	35553	28805	25838	24399	23693	23428	23765	25653	92184	137922
	27600	46643	35488	28228	24894	23731	23077	22829	23309	25650	98067	137112
RFB	25962	26472	25286	25368	24954	25283	25375	25624	26289	27633	92407	139187
	27199	26487	25919	35817	26969	25091	25329	25206	26248	28409	87044	146163
VAN	27558	25054	23660	23439	23607	23207	23082	34742	23392	111709	129406	152494
	27076	24583	23461	23837	23534	23562	23545	23763	23854	115120	142173	140474
	117517	151701	160098	138773	137032	133594	139927	136809	138216	142153	137826	143790
	27669	27956	27525	27110	27316	26809	26826	26755	27315	26909	27036	26663
LZD	28481	29118	25731	24114	24074	23639	23847	24230	27594	93726	106886	133842
	26322	28081	25406	23955	23505	23070	22855	23739	27247	93778	107841	142023
FOX	28023	116460	66177	43113	33547	30829	37857	107011	120569	149406	125205	136738
	27738	113573	64832	43448	33143	28882	25981	106109	110943	160830	145899	149753
AMK	27444	30345	26919	24979	24177	56173	109583	131321	143696	156033	126584	153450
	27205	30297	26939	25325	25030	104187	108047	122559	138352	130205	118130	140165
	130050	130855	130816	133885	128553	148645	140865	139267	149522	159938	146604	146982
	28435	28609	26824	28000	27636	26167						
LEV	28153	27598	24210	23203	23590	24059	23987	24975	60643	94725	120089	125450
	28523	26936	24777	24019	22984	23284	23124	23720	60545	89009	106819	144978
TGC	29014	55679	41311	33951	29244	26104	25336	26839	54832	92465	110702	150429
	30335	55909	40951	33543	29621	26970	25648	24140	47367	91793	91815	161803
							144600	137034	148864	149648	160658	126686
	29978	28922	27051	28200	27991	28314	27936	28116	28092	27738	27364	27628
EMB	30405	34791	29434	27917	27166	36827	85456	111177	111809	118320	114809	140964
	30408	33726	29802	27413	26746	35104	91215	102979	106774	109490	117221	138691
AZT	41148	26390	110584	108622	117536	110568	118516	115540	131117	134701	138125	277452
	42216	27288	99674	122061	105667	101596	113120	124942	134027	131879	152398	219318
	280821	274707	279411	268334	274308	298575	129535	130341	134397	96797	133110	145882

Rifabutin concentration range: 0.06-32 μg/mL

UbiA^{A260T} (S)

	27495	27700	26889	26916	26924	26752	26664	26543	27044	27203	27238	25624
RIF	28024	46190	34845	27347	25445	24600	24459	39012	119567	129729	117916	130704
	27936	44895	34484	28021	25005	24387	23980	41833	113618	118594	126012	129834
RFB	27355	19556	21113	22326	22442	22830	22598	23615	23802	26532	132617	178467
	27478	19112	21058	21581	22155	22408	21373	22373	23378	26686	109991	131829
VAN	26849	25029	23936	23654	23600	22381	21852	22748	93955	133724	129875	137627
	26767	25282	23970	23752	24238	23676	23610	24160	105154	125101	149716	135250
	132577	120640	125543	128177	183026	147992	126452	133736	136736	128791	141067	189158
	28489	27237	27228	27028	26733	26834	26666	26863	26853	26952	27153	27640
LZD	28423	28708	25812	24462	24687	24975	26030	34887	104014	98303	102181	135582
	28340	28291	25727	24611	23655	23878	25083	32574	105631	106879	128351	151931
FOX	28116	117142	66062	43910	34051	29132	40299	138523	140569	139051	146234	125464
	28459	112082	64341	43081	33447	29211	35287	129551	136164	135442	137870	132225
AMK	28009	30572	26966	25464	24795	150480	114426	113435	120668	144148	136819	129866
	28137	30680	26359	25299	24837	67177	98670	120477	133499	148754	186294	132746
	129690	143993	148757	177538	176871	160503	152177	190142	174908	145823	146935	186160
	29036	29004	27612	27768	27529	27824						
LEV	29089	26452	24407	24043	23635	24058	23596	25255	64947	111205	120147	182780
	27844	26322	24661	24021	23411	23137	22864	23769	57207	107192	122398	175636
TGC	29432	51996	39672	32217	28145	26020	25843	39612	80652	112716	113941	150754
	30087	51408	39697	32844	27804	27283	25716	38393	83140	120019	117357	157920
							160919	154614	136994	137437	157566	158405
	29650	29339	28874	27039	28373	28222	45149	39097	36320	37646	47264	35031
EMB	29761	30447	26270	25229	26153	67542	136171	117827	101987	124058	112083	181629
	29940	30162	27033	25595	25318	36631	111335	128665	106012	105708	106645	140812
AZT	43843	33473	132573	211965	197010	135730	214792	180978	202702	286898	272299	309874
	41422	33598	110984	212133	126725	261236	213970	245443	247226	274292	278219	316997
	260218	290840	325550	314033	321521	322152	178610	134060	148256	128581	138685	160569

Azithromycin concentration range: 1-512 µg/mL

UbiA^{WT} (R)

	123594	118734	113020	108350	108541	107016	107557	109757	112193	117422	118594	121879
RIF	115530	126608	88082	64115	53642	53081	55713	76060	675028	1029772	1192269	1260754
	107803	117701	86664	60235	48741	48553	52338	63180	734183	956315	1052874	1266260
RFB	97149	38545	43348	45016	49272	51550	56892	63420	71173	860222	1158572	1283886
	96631	37092	41734	43702	48211	51320	55642	60496	68405	782663	919944	1262454
VAN	109961	67412	65246	64720	65726	66461	67013	68124	70145	462417	913772	1278857
	114732	70020	66159	64216	67143	67698	66367	66009	69191	293684	891279	1160783
	1152816	1011197	917602	893963	884337	901727	887662	893760	916181	927132	1030190	1284050
	118093	110220	107919	105243	105815	104735	103781	104835	105918	108054	110318	120755
LZD	110408	95497	98968	94530	93465	93745	96024	113558	722225	1231257	1229596	1253743
	111278	94509	96766	94352	93416	92730	95540	108846	679535	1215141	1199723	1255620
FOX	109578	245527	181934	129181	111443	101667	97769	839249	1153346	1124009	1116808	1239488
	109520	239555	176596	126734	108398	99261	96747	890897	1147549	1125637	1156842	1274041
AMK	110816	189887	135236	112525	98966	155977	1074535	1179920	1186448	1119938	1152186	1273402
	112617	188901	137067	114262	104086	101612	387182	1170477	1171955	1155461	1184266	1288006
	1277962	1259848	1252926	1234812	1250854	1244247	1250128	1249622	1244144	1256185	1292191	1239077
	128837	120928	116861	115446	116129	115566						
LEV	121779	109666	106387	102970	100568	97487	100797	101562	887342	1232708	1223364	1325941
	120794	110375	108679	105805	99576	96855	95840	98625	838800	1181326	1216365	995223
TGC	120251	125490	151732	185669	149790	124212	108907	143666	939026	1239050	1241529	1257213
	123530	125046	154781	178871	144888	124025	111114	178011	1013369	1252565	1218568	1254618
							1228196	1300122	1291847	1349088	1307932	1310739
	134145	123867	120751	119005	119554	119134	118841	120806	123776	125130	125472	127971
EMB	128191	111455	113953	112192	183190	1215939	1212849	1195286	1206347	1229891	1238206	1310662
	128241	114605	115052	113353	155924	1135559	1188593	1154576	1177067	1199744	1208506	1292428
AZT	269154	139779	516454	1020407	950636	830272	807484	645906	671891	539092	485968	384801
	267449	145128	560284	1030214	944365	843079	790090	722045	559202	578128	569620	382523
	340915	379638	376438	367763	437923	462364	1287976	1273563	1266442	1294448	1307681	1172265

UbiA^{L39S} (R)

	120400	122040	119927	117191	117100	115642	115201	116268	117674	121309	122721	128528
RIF	123267	154461	110312	75712	63418	65128	65575	72310	92794	349459	1251708	1327319
	118110	149225	104801	68191	53686	51975	55192	65013	89584	403792	1145359	1278881
RFB	118509	45010	48642	47431	50426	53817	60946	67767	81240	102123	529453	1292286
	118254	47529	45074	45670	52052	54540	58096	64115	70816	94878	459150	1258538
VAN	118193	86155	78592	68752	69461	69875	70987	74722	77790	85407	1205121	1301179
	119154	86456	77540	72138	72680	72599	74142	77382	80612	87284	230185	1310184
	1338695	1263931	1197136	1190497	1150109	1159580	1190685	1218388	1223502	1259997	1343659	1332080
	125061	119811	119850	118595	118664	117744	117250	117463	119180	121715	123556	125395
LZD	123806	100042	104255	99557	99371	99299	102319	104762	156279	1209534	1305853	1276061
	118786	99093	101817	99179	94479	95455	98453	101568	182398	1156645	1244639	1335497
FOX	119404	270200	186782	137884	116566	103480	99800	100604	920470	1182806	1228688	1199837
	118810	267068	177559	132386	112772	104260	100683	104925	995852	1170084	1209282	1251421
AMK	117608	201754	143480	118322	107502	100387	107057	1167484	1143144	1160778	1171718	1244153
	116760	211702	144690	117556	114492	102521	102918	1176489	1223887	1140211	1229105	1247369
	1339442	1332105	1305239	1216243	1274104	1259839	1304023	1291524	1261888	1273984	1347376	1282826
	136396	133983	128836	127034	128874	130343						
LEV	134927	118999	114799	109853	105824	104965	105691	108815	139201	1159255	1275803	1337905
	129152	114093	111427	106438	102163	98025	99738	101615	120215	1097773	1202228	1293848
TGC	131685	123360	142249	179039	151756	126635	116545	155740	870131	1206808	1201934	1314615
	133006	119229	139164	184996	152507	125898	115464	147490	964684	1238394	1210587	1328396
							1265161	1346144	1308887	1295733	1280200	1243118
	136672	134912	132997	132287	132573	136520	257689	273429	266710	271568	264995	239634
EMB	140021	119112	120292	116637	116795	249867	1134928	1220077	1136120	1268648	1196879	1296526
	140000	120303	118630	114175	111398	218966	1079382	1191119	1173329	1174321	1228045	1316379
AZT	282464	144877	684831	1061199	975321	924751	913197	865194	845771	868066	787018	729668
	286873	147549	681675	1096050	999230	918443	915410	904113	892817	744515	837915	625023
	823198	974417	935861	967814	983144	891665	1252596	1372141	1221704	1320069	1350319	1253200

UbiA^{V57A} (R)

	144653	134874	127793	118743	118386	123627	114132	109201	111836	115665	123893	139911
RIF	139826	137813	92449	61909	48955	48461	52916	61923	96833	879023	925836	1149528
	132818	124288	87659	56199	45105	44997	50296	57575	105806	793456	813443	1013131
RFB	127809	41918	43651	43096	45061	49008	56545	58631	72841	112287	803620	1021411
	138478	44269	43168	43802	48671	62913	57827	63585	69114	129138	826824	1236874
VAN	141559	96054	76596	72541	72926	73652	73643	70671	73220	82976	965517	1296732
	134342	96227	84734	81593	80971	77717	76589	75918	81466	88915	1064719	1312442
	1242355	1280184	1189460	1105261	1174574	1293088	1237880	1133433	1152704	1181720	1262321	1304811
	131700	117488	113400	109842	108292	107347	105734	105196	106419	109913	114723	129081
LZD	124654	110225	107093	102891	101090	99875	100688	108248	306829	1166533	1200319	1174487
	120899	106134	105987	101458	97021	99079	98688	101372	258580	1160327	1188752	1218387
FOX	124344	252358	188650	132740	113004	106367	102116	255755	1053007	1089888	1177157	1192086
	124648	248212	186598	134318	118958	105185	98839	247685	1076631	1084292	1209996	1284423
AMK	126219	213850	151476	124815	111240	100749	97091	1130556	1082465	1142658	1199679	1267401
	128711	217702	154158	126570	118718	105165	100682	1133268	1158740	1199785	1239819	1338624
	1251819	1298541	1280507	1269372	1148359	1269135	1280407	1271154	1236704	1262848	1318341	1312145
	153801	140874	134553	132606	130898	130563						
LEV	151033	137331	133957	128351	122687	120795	118958	122992	190582	1190527	1251572	1277129
	147325	137123	134526	128177	120728	118667	116778	118959	251220	1164175	1210963	1258044
TGC	151405	125899	146149	191389	158358	133832	124486	240643	1027967	1192167	1115610	1276211
	159579	126104	151311	189679	163832	138368	125402	196904	908303	1151895	1203526	1285685
							1219405	1238342	1221224	1222743	1241493	1289497
	183172	153167	142300	140971	141610	141022	253353	259652	264354	264888	271772	274470
EMB	161980	140526	135938	130241	138342	484800	1247755	1205957	1212600	1197157	1223579	1246824
	159591	140766	140520	132959	135695	435897	1224236	1207509	1182882	1183980	1178542	1261324
AZT	282699	152432	182255	991928	1004673	891510	885441	821429	808928	762052	841396	382299
	283712	151172	189121	973946	999623	930698	893274	858628	817093	386186	554525	793333
	332001	639756	705358	434703	682884	728635	1271084	1273808	1211042	1175966	1276092	1200035

UbiA^{T216P} (R)

	133795	128878	123547	121240	121150	120736	121576	121361	124297	125543	129552	136930
RIF	131885	147382	102872	75474	62303	63004	68577	82126	407566	1185410	1193140	1213386
	130093	146137	102943	74136	61210	60902	65585	79265	223832	1148380	1168946	1230521
RFB	130028	50907	56929	60686	65946	72340	81501	90150	100895	905503	1052968	1209188
	129526	50881	56787	60905	64879	72643	80753	91704	102132	780671	1064787	1218694
VAN	129645	111312	108388	107538	105420	107222	106212	108350	110248	660819	1243435	1236393
	131999	111377	110658	107021	109059	108129	110534	111468	112294	997943	1275701	1241511
	1092190	1254188	1263449	1188171	1243996	1239065	1236269	1260471	1252713	1270361	1269158	1282610
	132599	127367	122500	120195	118872	118957	116553	118993	118692	121246	123755	132361
LZD	130085	122894	112382	106150	106716	106686	107884	119242	554915	1108745	1238664	1232968
	127840	124791	111027	105174	101919	102757	101899	112512	221781	1089390	1157924	1212866
FOX	127116	244483	191113	138178	119669	115404	401642	1061705	1226559	1207223	1201844	1241324
	125013	236990	185135	142929	116910	112096	481509	1080920	1221869	1198261	1211638	1225189
AMK	125224	211790	153405	129353	115612	113773	431011	1210490	1220463	1204904	1205556	1250844
	126209	207377	157554	129171	119319	115478	334461	1222616	1227280	1232040	1228500	1292894
	1354277	1275744	1108185	1230447	1250887	1258506	1099007	1248387	1229417	1271544	1302990	1316598
	148924	144934	139747	141137	142035	142788						
LEV	146257	140441	135869	127973	123184	120656	123570	125480	600817	1225897	1242632	1289878
	145151	135033	135989	126502	118908	115607	116713	119789	605127	1160479	1199014	1232859
TGC	144369	116706	138508	180175	156459	135026	125899	261244	1031476	1184519	1177734	1252782
	145815	116406	141897	180091	152676	132340	126364	254544	1043599	1178481	1190403	1270949
							1307352	1306369	1247634	1269699	1294208	1237434
	156257	150748	148246	144734	148791	152733	272955	276322	269740	276885	270828	268615
EMB	156659	143058	141034	135535	135977	772076	1265852	1233778	1254793	1225643	1086529	1138763
	154289	143910	142372	133889	133871	681939	1173124	1168852	1191784	1189771	1227301	1185751
AZT	293036	153982	613900	933922	825870	716014	652192	615331	598769	703072	734034	754133
	301087	158780	348229	945030	835704	704803	689934	584469	564131	602594	668509	868487
	309143	539726	685147	691121	805322	742291	1312024	1322098	1287474	1293250	1317430	1295417

UbiA^{A260T} (R)

	140180	133066	125691	124951	121952	121548	124105	122710	125727	126614	130532	140191
RIF	136076	158079	108680	78906	66845	67335	74937	86714	274997	1223680	1197644	1276163
	134376	157910	97659	80055	66254	65852	72353	84113	210321	1214617	1178976	1260906
RFB	135784	55795	61628	65040	69514	79138	88458	90461	100107	612858	1164899	1257144
	133303	53268	60385	66409	69102	75201	85704	91308	100501	511544	1180660	1251267
VAN	134643	112157	107480	105810	103862	106711	108551	106185	107794	135434	1199518	1264448
	137196	115404	110907	109525	109268	109932	111115	110840	111667	122488	1279231	1303615
	1243014	1281521	1332237	1323325	1318008	1336912	1296743	1323256	1303682	1323077	1322231	1266722
	133732	123337	119581	115351	115493	115296	114467	113537	115903	118531	121941	132846
LZD	127411	127947	109011	104833	103202	100957	101304	106260	222630	1241364	1191376	1259182
	125991	124978	113764	105790	99407	98149	97981	106426	258600	1177000	1165449	1221472
FOX	127025	262770	190077	134447	115456	107393	104534	238310	1083837	1142653	1130880	1261520
	128706	257755	187031	135210	113432	105317	101976	301399	1138723	1124727	1145202	1261683
AMK	127565	210298	150431	124551	110119	104427	100063	1054885	1152241	1138074	1149433	1285422
	131368	223806	158013	129202	116130	108139	192791	1249516	1185135	1196746	1197619	1304100
	1273523	1271541	1302611	1333355	1331621	1336987	1321711	1321932	1296684	1302239	1312183	1276325
	138489	127889	124850	123507	124190	125355						
LEV	133745	120495	117930	111577	108046	107989	108914	112599	157059	1256767	1302176	1305412
	133210	116268	117520	112470	106412	104696	106134	107766	134162	1198527	1231384	1276422
TGC	135692	116700	141233	184922	158705	132891	120296	181887	906337	1223503	1208455	1278071
	140391	119501	144223	186072	157599	133549	122373	179984	940221	1232236	1184327	1270036
							1331051	1325267	1285310	1261618	1254607	1117906
	149350	140722	137085	135162	135916	137723	259729	262920	266301	271270	273599	279853
EMB	142021	123024	122594	118598	117648	236832	1268283	1256422	1248186	1232241	1222998	1310299
	142307	121714	121882	118049	115714	230285	1208340	1192210	1205107	1180957	1183635	1267744
AZT	293033	144363	1013044	963427	835569	664900	623020	564685	561639	654373	545673	741192
	296743	149035	1048586	966828	799672	638556	582125	590457	541618	632177	584153	688608
	232950	377449	422467	465841	484642	482725	1281025	1336229	1284728	1248708	1308024	1195095

<u>Table S3</u>: LC/MS analysis of the unmodified and covalently modified oligoarabinosides released from the LAM of smooth and rough *ubiA* mutants upon endoarabinanase digestion. Shown in bold letters are the relative percentages of total (including, unmodified and covalently modified) Ara_4, Ara_5 and Ara_6 oligoarabinosides released upon *Cellulomonas gelida* endoarabinanase digestion of LAM from the different strains. The individual representation (expressed as percentages) of unmodified and covalently modified oligoarabinosides within each group are below the bolded rows. This analysis represents one technical replicate of one biological replicate (same LAM preparation as analyzed in Table 2).

	**Smooth**					**Rough**				
Strain	WT (S)	L39S (S)	V57A (S)	T216P (S)	A260T (S)	WT (R)	L39S (R)	V57A (R)	T216P (R)	A260T (R)
**Total Ara_4**	**25.9**	**24.7**	**24.6**	**17.9**	**20.7**	**25.4**	**23.2**	**20.7**	**16.1**	**16.0**
Unmodified Ara_4	65.6	68.7	69.0	78.7	39.6	48.1	70.3	67.0	52.7	58.0
Ara_4 + succinate	28.7	25.1	26.7	21.3	16.9	10.7	0	12.0	7.0	0
Ara_4 + acetate	2.5	6.2	0	0	24.4	19.2	19.0	11.8	20.1	20.3
Ara_4 + acetate + succinate	3.2	0	4.3	0	19.1	21.9	10.7	9.2	20.2	21.7
**Total Ara_5**	**43.0**	**39.7**	**41.5**	**36.6**	**33.9**	**33.7**	**41.2**	**40.6**	**50.1**	**36.4**
Unmodified Ara_5	68.0	74.4	67.6	70.4	54.7	67.2	69.1	72.9	41.7	67.1
Ara_5 + succinate	26.5	25.6	32.4	29.6	29.7	19.2	26.1	24.5	51.4	26.1
Ara_5 + acetate	4.0	0	0	0	6.5	5.0	0	0	3.2	0
Ara_5 + acetate + succinate	1.5	0	0	0	9.1	8.6	4.7	2.6	3.7	6.8
**Total Ara_6**	**31.1**	**35.6**	**33.9**	**45.6**	**45.3**	**41.0**	**35.6**	**38.7**	**33.7**	**47.6**
Unmodified Ara_6	68.9	69.4	63.9	69.5	72.8	78.5	70.3	64.9	74.6	80.4
Ara_6 + succinate	31.1	30.6	36.1	21.6	19.0	18.0	21.5	24.8	19.1	19.6
Ara_6 + acetate	0	0	0	8.8	8.2	3.5	8.2	10.2	6.2	0
Ara_6 + acetate + succinate	0	0	0	0	0	0	0	0	0	0

<u>Figure S1</u>: Predicted impact of the fourteen UbiA mutations on the binding affinity of DP and PRPP. Mutants that reduce the binding affinity of DP comparatively more than PRPP and vice versa are shown in blue and red, respectively. Mutants with the largest deviation from the x=y line are labeled in red (L187F) or blue (D44G, D259G) along with the four mutations under study therein. The datapoints of V57A and V285A overlap in the plot as they share the same impact on the binding affinity of DP and PRPP.

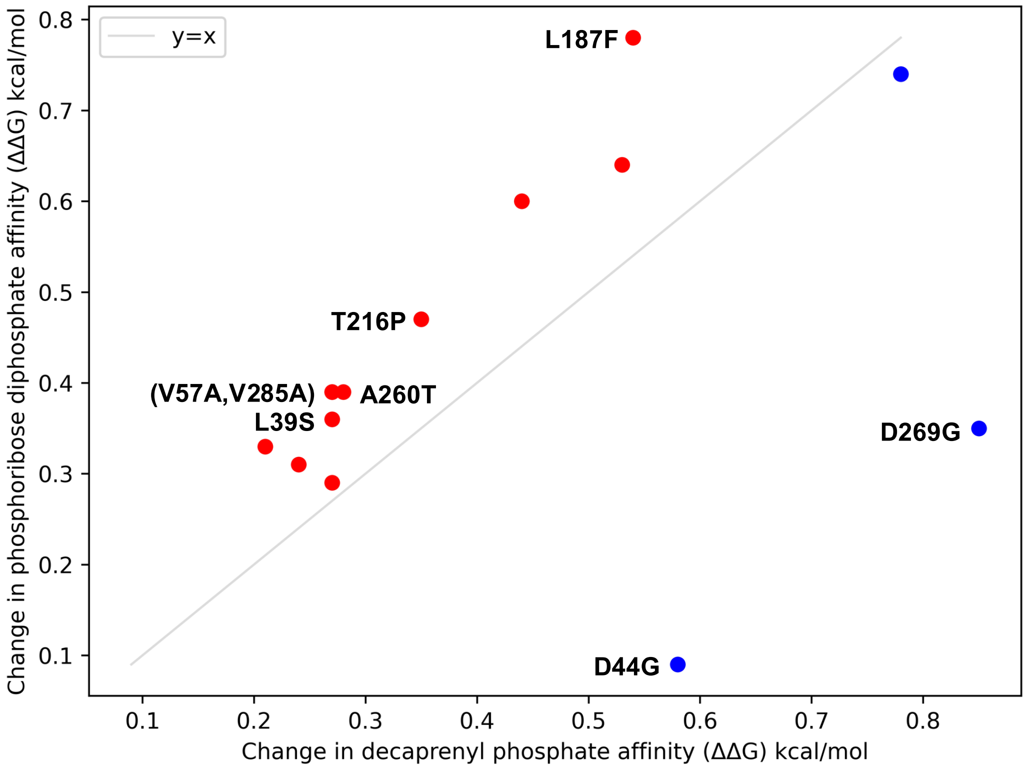

<u>Figure S2</u>: Allelic replacement at the *ubiA l*ocus of rough and smooth merodiploid *M. abscessus* ATCC 19977 strains.

Genomic DNA was extracted from the WT parent *M. abscessus* ATCC 19977 strains and corresponding rough and smooth *Mabs*Δ*ubiA*/pMV306-*ubiA*^{WT} strains, and PCR-amplified using primers *ubiA* KO Fw (5'- GCCGGAGCACATCGCCTCCAT – 3') and *ubiA* KO Rv (5' – CGCCCTGGCGGATCCAGTACA – 3') to confirm allelic replacement at the *ubiA* locus of the merodiploid strains. The expected sizes of the PCR fragments in the WT and mutant strains are indicated below the gel.

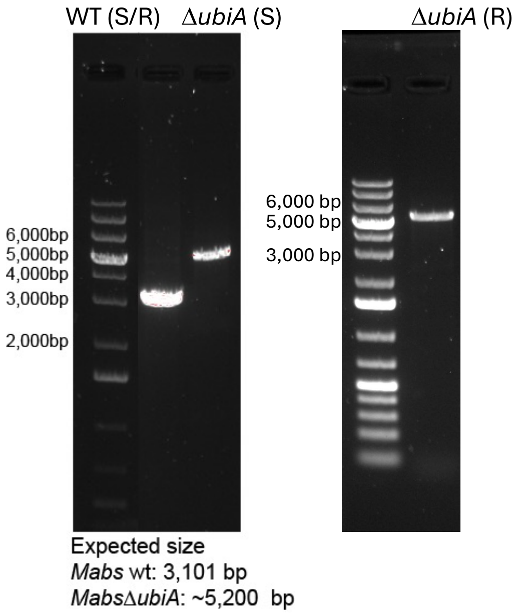

<u>Figure S3</u>: Impact of patient-derived *ubiA* mutations on the distribution of mycolic acids between the inner and outer leaflets of the outer membrane of *M. abscessus*.

Smooth (A) and rough (B) WT and *ubiA* mutant strains were radiolabeled with [1,2-^{14}C]-acetate and incubated for an additional 4 h before the cells were collected for total lipid and cell wall mycolate extraction and analysis. Radiolabeled lipids were analyzed for TMM and TDM content on silica gel 60–precoated TLC plates (Millipore Sigma) eluted with chloroform:methanol:water (20:4:0.5, by vol.). Radiolabeled cell wall-bound mycolic acids were prepared from delipidated cells and derivatized to mycolic acid methyl esters (MAMEs) as described previously (Etienne *et al.*, 2002). Radiolabeled mycolylated products were visualized using a Sapphire Biomolecular Imager and quantified using the AzureSpot analysis software (Azure Biosystems).

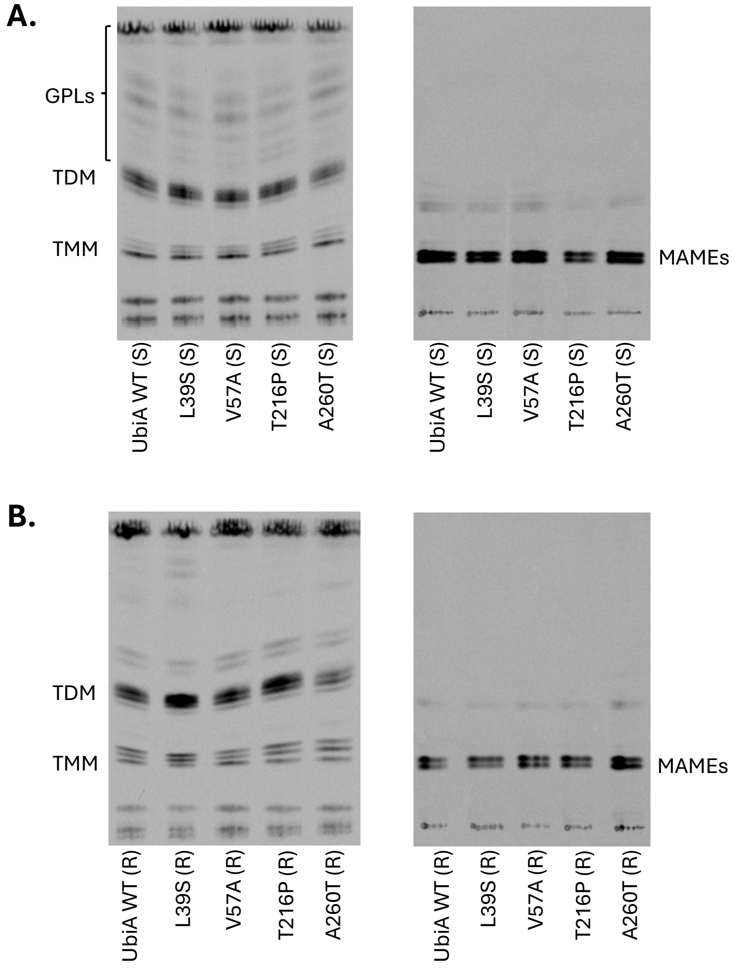

Etienne, G., Villeneuve, C., Billman-Jacobe, H., Astarie-Dequeker, C., Dupont, M. A., and Daffe, M. (2002) The impact of the absence of glycopeptidolipids on the ultrastructure, cell surface and cell wall properties, and phagocytosis of *Mycobacterium smegmatis*. *Microbiology (Reading)* **148**, 3089-3100.

<u>Figure S4</u>: Growth of smooth (A) and rough (B) *ubiA* mutant strains in 7H9-ADC-Tween 80 and SCFM at 37°C.
The results presented are representative of two independent experiments for each morphotype and each medium.

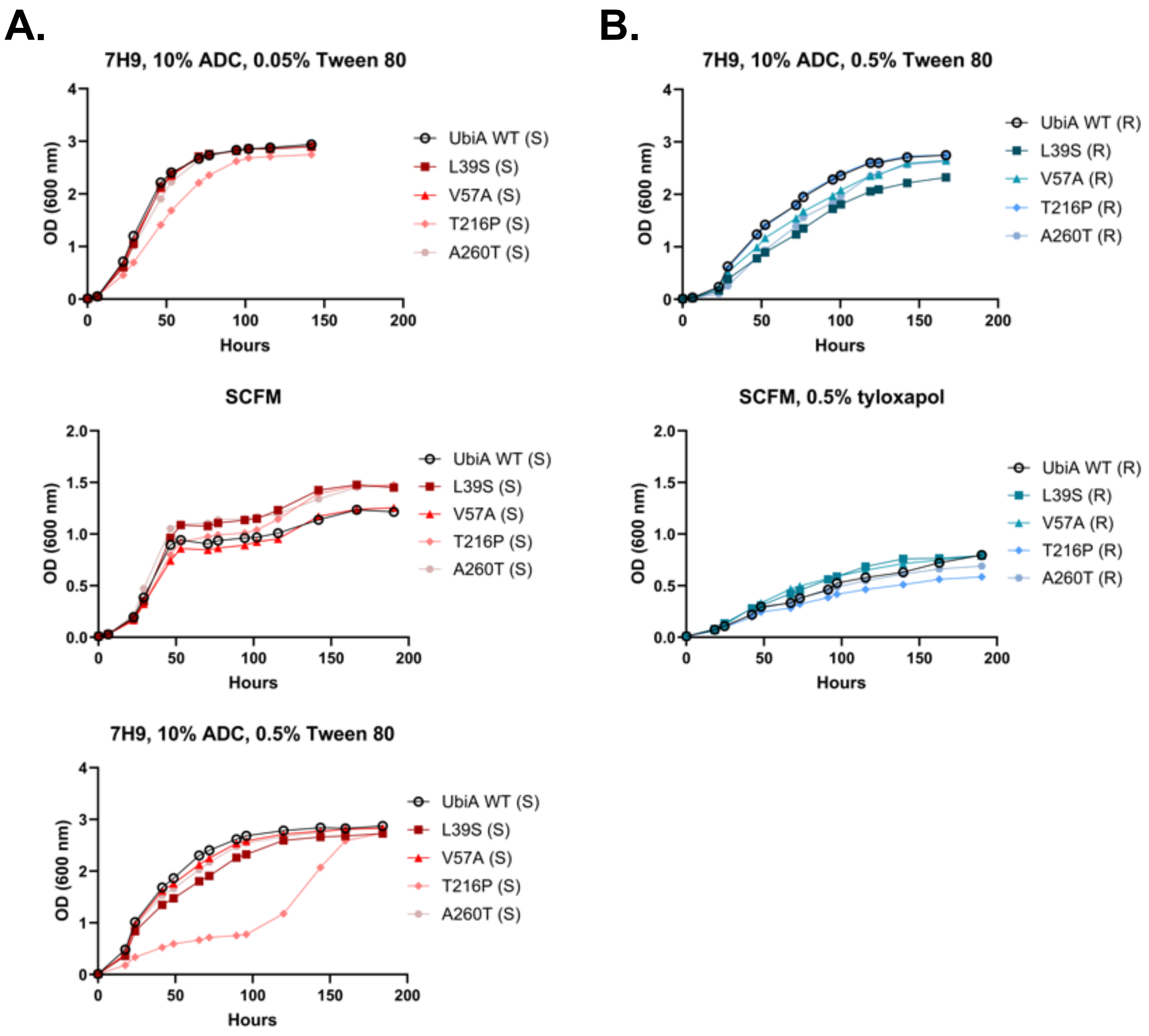

<u>Figure S5</u>: Impact of patient-derived *ubiA* mutations on the morphology and surface hydrophobicity of smooth and rough *M. abscessus*.
(A) The colony morphology of S and R mutants was compared to that of their respective control strain UbiA^{WT} (R/S) by plating cultures of each strain on 7H11-OADC agar and incubating the plates for 10 days. Scale bars = 1 cm. (B) Congo Red binding on a Tryptic Soy agar. Shown are the average ± SD absorbances for triplicate cell pellets for each strain. A one-way ANOVA with Dunnett's multiple comparisons test against UbiA^{WT} (R/S) was performed (* p<0.05).

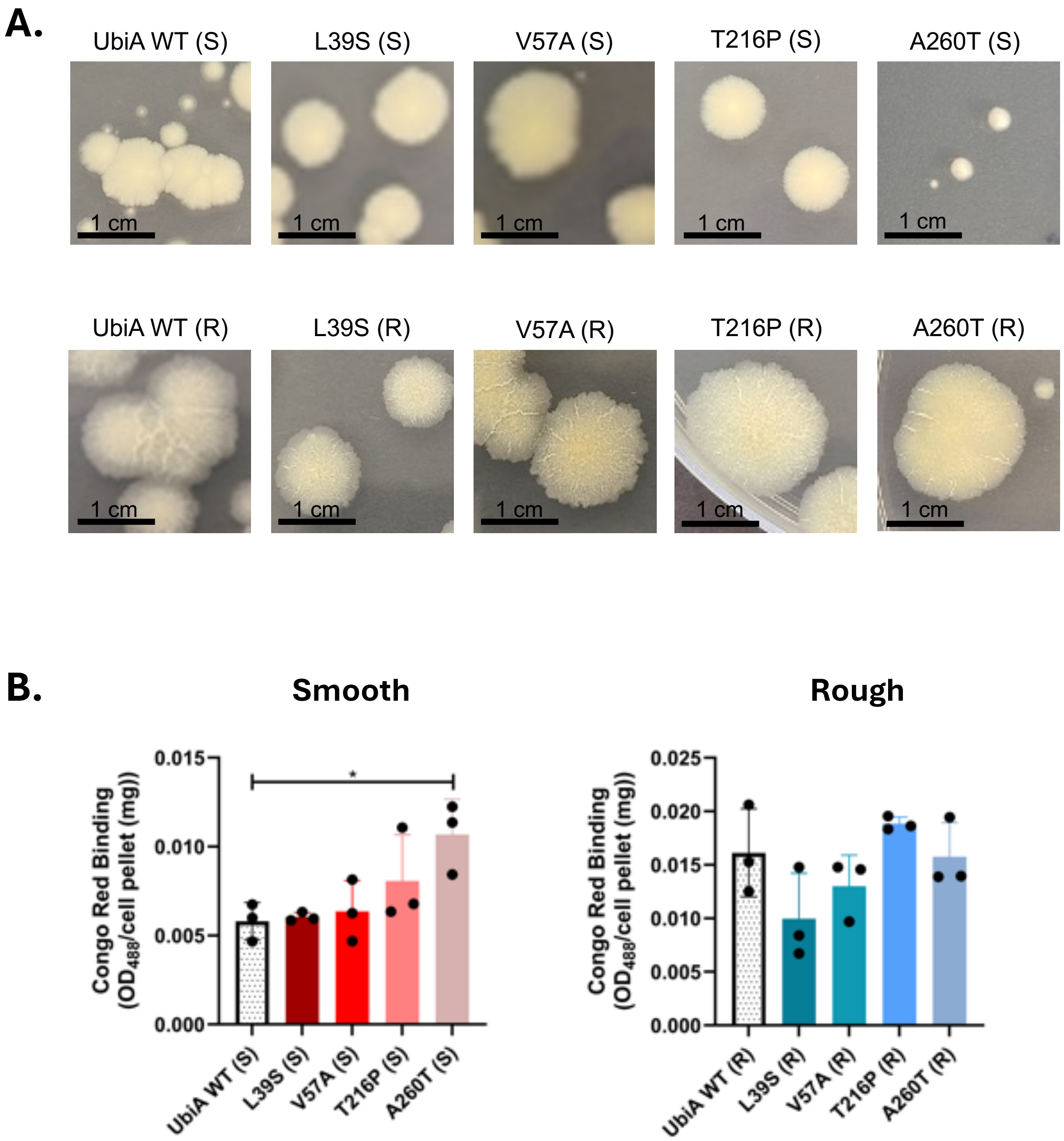

<u>Figure S6</u>: *ubiA* mutations do not alter the ability of rough *M. abscessus* to form cords.
The rough morphotype control and mutant strains were inoculated in Tryptic Soy broth at a concentration of 10^4 CFU/mL and their ability to form serpentine cords was compared after 3 days of incubation at 37°C. All assays were performed as described under Materials and Methods. The yellow scale bar in the bottom-right is equivalent to 500 µm.

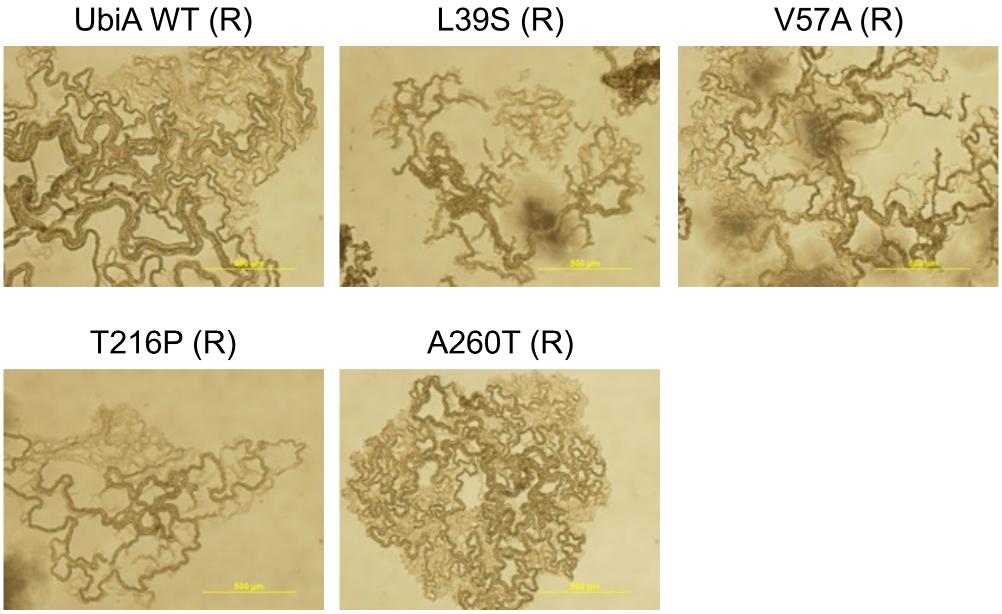

<u>Figure S7</u>: NF-κB activation in HEK-TLR2 cells by purified LAM from rough *M. abscessus* control and *ubiA* mutant strains.

Activation of the TLR2-NF-kB axis by LAM purified from rough *M. abscessus* control and *ubiA* mutant strains (1 µg per well) was assessed using the reporter cell line HEK-Blue-hTLR2. Shown are averages ± SD of absorbances measured at 650 nm after 24 h of incubation in triplicate for each lipoglycan preparation, and the results are representative of two independent experiments. Purified LM (1 µg per well; BEI Resources NR-14850) and purified LAM (1 µg per well; BEI Resources NR-14848) from *M. tuberculosis* H37Rv were used as controls for TLR2 activation. A one-way ANOVA Dunnett's multiple comparisons test against LAM purified from UbiA^{WT} (R) was performed (*** p<0.001; **** p<0.0001).

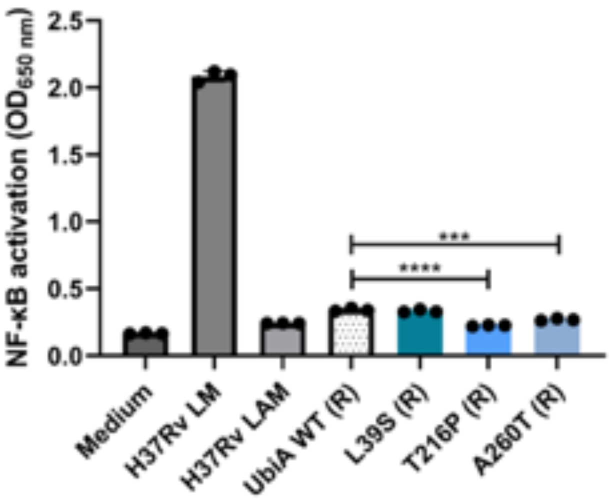

<u>Figure S8</u>: Intracellular replication of *ubiA* mutants in human monocyte-derived THP-1 macrophages and A549 epithelial cells.

(A) THP-1 macrophages were infected at an MOI of 1. Intracellular replication (CFU/mL) was quantified from 2 to 72 h post-infection for S mutants and from 2 to 48 h post-infection for R mutants. by CFU plating. Shown are averages ± SD for triplicate wells for each strain. Results are representative of two independent assays for the S strains and of three independent assays for the R strains. A two-way ANOVA Tukey's multiple comparisons test against UbiA^{WT} (R/S) was performed for each time point (* p<0.05, ** p<0.01).

(B) A549 lung alveolar type II epithelial cells were infected at an MOI of 1 for both morphotypes. Intracellular replication was quantified from 2 to 72 h for S strains and from 2 to 48 h for R strains by CFU plating. Shown are averages ± SD for triplicate wells for each strain and results are representative of three independent assays for both morphotypes. A two-way ANOVA Tukey's multiple comparisons test was performed against UbiA^{WT} (R/S) for each time point (* p<0.05, ** p<0.01*** p<0.001; **** p<0.0001).

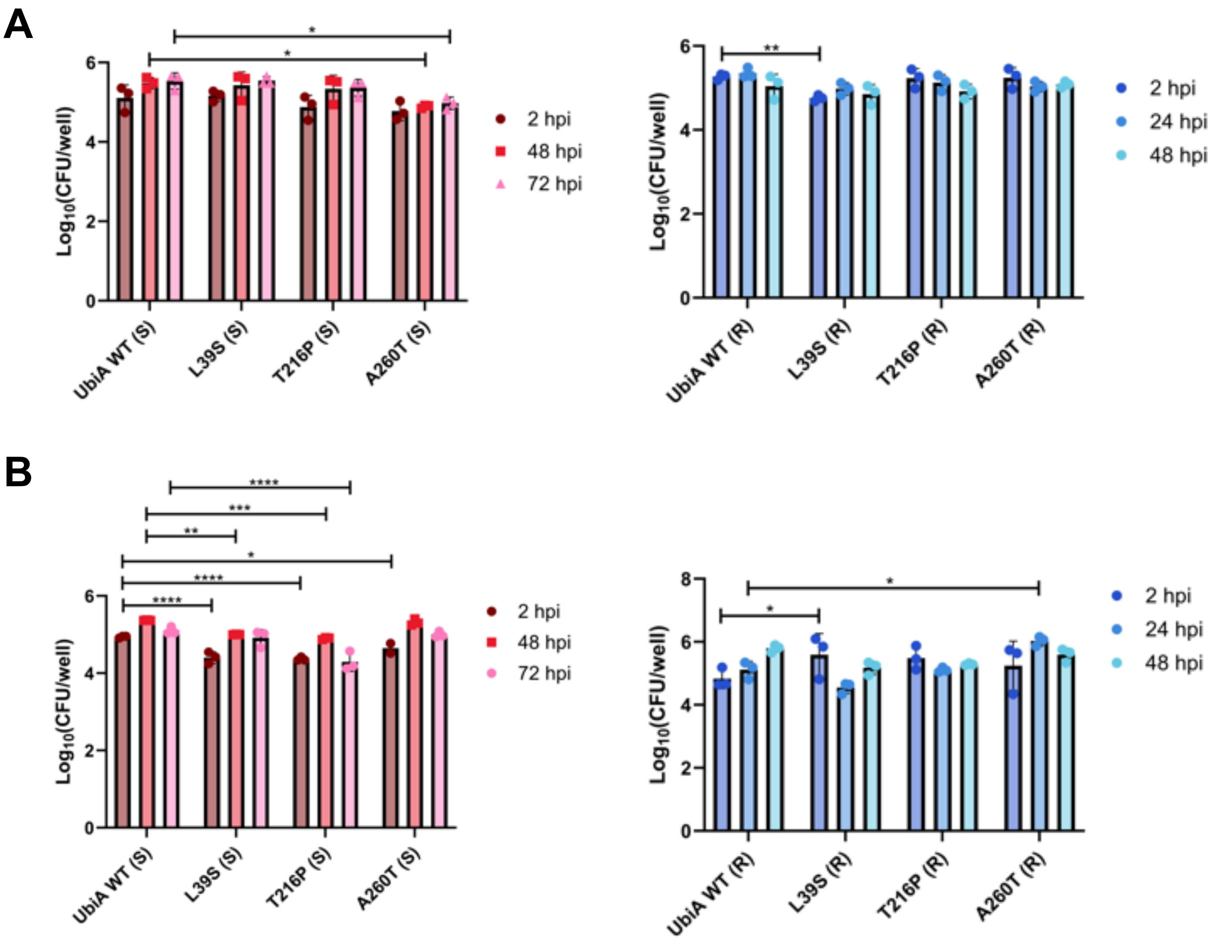

<u>Figure S9</u>: IL-8 secretion induced by the control and *ubiA* mutant strains in human A549 epithelial cells.

A549 epithelial cells were infected at an MOI of 1 for both morphotypes. IL-8 secretion at 48 h post-infection was determined by ELISA. The results presented are the averages ± SD of triplicate wells from one experiment and are representative of three independent experiments. No statistical difference was found neither in the S or R morphotype between mutant strains and their UbiA^{WT} (R/S) control per a one-way ANOVA Dunnett's multiple comparison.

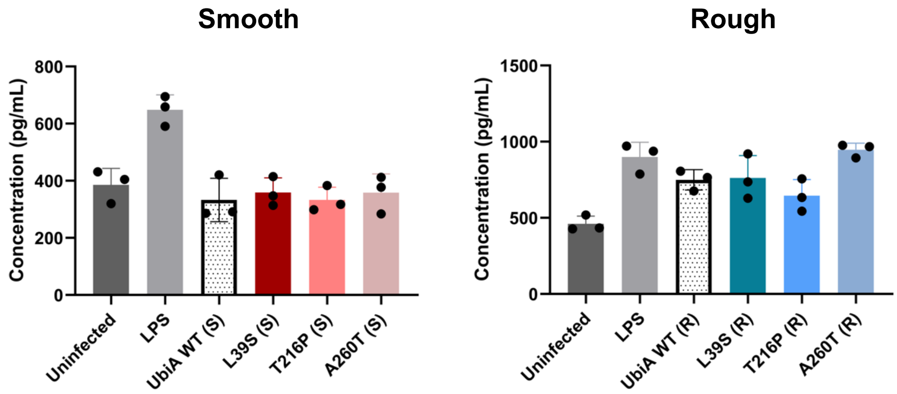

Supplement: Supplemental material — Tables S1 to S3 and Figures S1 to S9. [file mbio.00322-25-s0001.pdf]
